# The historical demography of the Martha’s Vineyard signing community

**DOI:** 10.1093/deafed/enad058

**Published:** 2024-01-29

**Authors:** Justin M Power, Richard P Meier

**Affiliations:** Department of Linguistics, The University of Texas at Austin, Austin, TX, United States; Department of Linguistics, The University of Texas at Austin, Austin, TX, United States

## Abstract

The deaf population of Martha’s Vineyard has fascinated scholars for more than a century since Alexander Graham Bell’s research on the frequent occurrence of deafness there and since Groce’s book on the island’s signing community (Groce, N. E. (1985). *Everyone here spoke sign language: Hereditary deafness on Martha’s Vineyard*. Cambridge, MA: Harvard University Press.). In Groce’s work, and in that of subsequent scholars, the Vineyard signing community has often been portrayed as remote and outlying, having developed independently of mainland signing communities for roughly 133 years until 1825. We re-examine that interpretation in light of historical, demographic, and genealogical evidence covering the period 1692–2008. We argue that the Vineyard signing community began in Chilmark in 1785, 93 years later than previously thought, and that it had had a brief period of independent development, roughly 40 years, before becoming well connected, through deaf education, to the nascent New England signing community. We consider the implications of the Vineyard community’s history for our understanding of how village signing communities develop.

## Introduction

In 1784, some residents of Chilmark had never encountered a deaf individual. Yet, just a half-century later, a signing community would flourish in this small town on the western end of Martha’s Vineyard. In the early 1840s, four families, each with multiple deaf children, were interviewed in Chilmark by William Turner, an instructor at the American School for the Deaf (ASD) in Hartford, CT. Although Turner (1847, p. 28) referred to the location as “a small town in southeastern Massachusetts” and although he identified the parents of these families only by their initials and by the titles “Mrs.,” “Mr.,” or “Capt.,” we can confidently identify them by name (Groce, 1981). Lydia Mayhew was the mother of the first deaf child to be born into these four families; she gave birth to her son Benjamin Mayhew Jr. in February 1785. Lydia reported that she first saw a deaf person while she was carrying Benjamin. The other parents echoed her story; aside from Benjamin Jr. and the other deaf children born after him, they too had never met a deaf person prior to the birth of their first deaf child.

How could the reports of these women have been true, given prior accounts of the 18th century deaf population on Martha’s Vineyard? Groce (1981) discounted Turner’s report because of its focus on “maternal fright”—that is, the notion that a pregnant mother’s psychological reaction to deafness might itself be a cause of deafness. We will argue that, with respect to the absence of deaf individuals in Chilmark, the reports of these families are likely to have been fully accurate. We come to this conclusion by analyzing demographic data on the deaf population of Martha’s Vineyard; these data are now available in a variety of electronic and archival records. Our work contributes, we believe, to the growing literatures on village sign languages (e.g., Zeshan & de Vos, 2012) and on the sociolinguistic typology (Trudgill, 2011) of signed languages (Hou & de Vos, 2022; Schembri et al., 2018).

Although prior scholarship has cast the Martha’s Vineyard signing community as a remote, outlying community, we will argue that, by the mid-19th century, it was well connected to the nascent New England signing community and that its growth and subsequent decline were in part caused by that connection. Moreover, we will show that the history of the Vineyard signing community is interwoven with American history more broadly. Prominent figures from colonial American and U.S. history encountered members of the island’s deaf population. Judge Samuel Sewall, of Salem witch trials infamy, met one of the island’s first deaf residents; John Adams, who would later be the second American president, met another; Alexander Graham Bell’s fascination with the island’s deaf population led him to visit the Vineyard in 1885 and to interview a family in Chilmark with six deaf members; and Thomas Hart Benton, the 20th century American regionalist, created two paintings of deaf islanders, one now at the Martha’s Vineyard Museum and the other at the Whitney Museum of American Art. Groce’s groundbreaking work on the Vineyard signing community and its language (Martha’s Vineyard Sign Language, MVSL) painted a rich and important picture of the history of that community and of day-to-day life inside it. Here, we propose a revised account of its founding, growth, and eventual decline.

### Martha’s Vineyard

**Figure 1:**
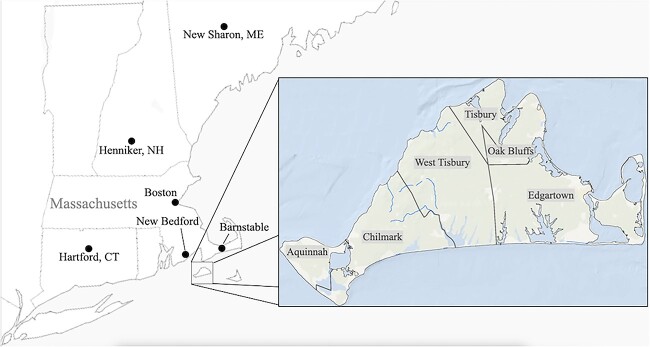
Martha’s Vineyard and other locations in New England mentioned in this article. The town of West Tisbury was incorporated in 1892 (Banks, 1911b). When discussing events prior to the town's founding, we use ``western Tisbury.''

Martha’s Vineyard is an island off the coast of Massachusetts; see Figure 1. English settlers arrived in 1642; they largely displaced the indigenous Wampanoag (Algonquian), who remain today in Aquinnah (formerly Gay Head), the westernmost community on the island (Banks, 1911a). On Groce’s (1985, p. 21) account, a substantial portion of the English residents had ancestors who had come from Kent; that Kentish population brought to the island a recessive gene for deafness that was the presumed cause of “Vineyard deafness.” Although significant African-American and Portuguese communities would settle on Martha’s Vineyard in the 19th century, deafness was restricted to the population of English ancestry.^1^

Why is work on the signing community of Martha’s Vineyard significant? (i) Prior discussions of Martha’s Vineyard—in particular the oral history interviews collected by Groce (1985), Lee (1998), and Nash (e.g., Bahan & Nash, 1995)—have contributed significantly to our understanding of how deaf and hearing individuals have interacted successfully in small-scale societies. (ii) The rich historical record will allow us to document, with great granularity, the emergence, growth, and decline of the deaf population on the island. This analysis will contribute not only to our understanding of the Vineyard signing community but also to our understanding of other village signing communities. (iii) Signers of MVSL have been seen as contributors to the emergence of American Sign Language (ASL). Deaf individuals from Martha’s Vineyard joined the New England signing community that formed with the founding of ASD in 1817 (Power & Meier, 2023). Thus, a thorough understanding of the deaf population on Martha’s Vineyard, and of its associated signing community, will contribute importantly to our understanding of Deaf history in the U.S. (iv) Lane et al. (2000) suggested that genetic factors—in particular, autosomal recessive deafness (as on Martha’s Vineyard) versus autosomal dominant deafness (as in Henniker, NH)—have contributed historically to the emergence of two types of communities, namely, *assimilating* and *differentiating* societies. The historical data we adduce here will allow us to re-examine this hypothesis. Lastly, (v) the signing community on Martha’s Vineyard has been embraced in the popular culture as something of a deaf utopia (Kusters, 2010).

### Village signing communities and their languages

The study of village signing communities and of village sign languages intersects with two primary research areas: language emergence and language typology—more specifically, sociolinguistic typology. Village signing communities have developed within relatively small-scale societies with dense social networks that include deaf individuals. These communities form within populations that are reproductively isolated, whether by geography (Martha’s Vineyard: Groce, 1985; Providence Island: Washabaugh, 1979; Ban Khor, Thailand: Nonaka, 2009; south central Turkey: Ergin et al., 2018), ethnicity (Ghardaia, Algeria: Lanesman & Meir, 2012), or marriage practices (Al-Sayyid, Israel: Sandler et al., 2005). Groce argued that carriers of a recessive gene for deafness were widespread in the relatively isolated population of Martha’s Vineyard. Frequent consanguineous marriage meant that many deaf children were born there. The island’s dense social network led to the inclusion of both deaf and hearing individuals in its signing community.

Village sign languages are a sociolinguistic class of signed languages that develop in such communities. These languages yield important insights into linguistic properties that can emerge over short spans of time (e.g., two to three generations) within signing communities that have little or no contact with other sign languages (e.g., Sandler et al., 2005). As such, the study of village sign languages forms part of a now-rich research tradition that seeks insights into language emergence by studying deaf children and deaf populations who have experienced an interruption in the intergenerational transmission of language (e.g., Goldin-Meadow & Mylander, 1998; Senghas & Coppola, 2001). Such interruptions are a consequence of the fact that most deaf children are born to hearing parents; Mitchell and Karchmer (2004) have estimated that as few as 4% of deaf children in the U.S. have deaf parents. In general, deaf children’s hearing levels mean that they have little access to the speech signal in the parental input. The fact that their hearing parents likely know no sign means that such children have typically not received systematic linguistic input in the language modality, the visual–gestural modality, that is fully accessible to them.

Sign language emergence has been examined over a range of scales, from the first-generation homesign systems of isolated deaf children (Goldin-Meadow & Mylander, 1990) to second-generation homesign systems (Neveu, 2019) and family sign languages (Horton, 2022; Hou, 2016), to larger village sign languages of varying time depths and community sizes (see the contributions in Zeshan & de Vos, 2012; de Vos & Nyst, 2018). The emergence of new national sign languages has also been tracked, both in Nicaragua (Polich, 2005; Senghas & Coppola, 2001) and in Israel (Dachkovsky & Sandler, 2009; Meir & Sandler, 2008). Recent work (Power & Meier, 2023) has reported a detailed quantitative analysis of the signing community in which ASL first arose; that community was formed by the students who attended ASD in Hartford in the years following the school’s 1817 opening.

As noted, village sign languages offer unusual opportunities to observe the emergence of new linguistic systems. Like homesign systems (Goldin-Meadow & Mylander, 1990), Al-Sayyid Bedouin Sign Language shows evidence of syntactic structure in its reliable word order tendencies (Sandler et al., 2005). However, there is scant evidence for phonological structure in that language; moreover, there is significant variation in vocabulary across different families (Sandler et al., 2011). The literature to date has suggested that some village sign languages show important typological differences from national sign languages, such as ASL. Unlike national sign languages, village sign languages typically lack a fingerspelling system or other means of encoding the written language of the local hearing community (de Vos, 2012b; but cf. Washabaugh, 1981, on Grand Cayman Sign Language). Linguistic devices such as directional verbs that are a ubiquitous means of marking argument structure in national sign languages appear to be absent from some—but not all—village sign languages; compare Kata Kolok (de Vos, 2012b) to Yucatec Maya Sign Language (Le Guen, 2022) and the village sign language used in San Juan Quiahije (Hou, 2016). Despite having emerged on the same time scale, Israeli Sign Language displays directional verbs, but Al-Sayyid Bedouin Sign Language lacks them; indeed, it appears to make scant use of space in its grammar (Sandler et al., 2005).

MVSL is one of the first village sign languages to have been identified—although its linguistics have been scarcely examined, in large part because the last deaf signer who may have known the language died in 1952. Groce (1985) argued that a sign language was brought to Martha’s Vineyard by English settlers and was used by the deaf and hearing members of this community. She has suggested that MVSL may have been an important contributor to the emergence of ASL, once students from Martha’s Vineyard began enrolling at ASD in 1825.

#### How do village signing communities form?

A sizeable number of village signing communities have now been identified (Zeshan & de Vos, 2012); all are reproductively isolated. A gene for deafness must be present in a population at its founding, must be introduced from the outside through in-migration, or must arise through mutation. Consanguineous marriage increases the likelihood that deafness will be expressed in the population. There are various genetic bases for deafness in these communities: Most frequently, deafness seems to be a non-syndromic recessive trait (Martha’s Vineyard: Groce, 1985; Al-Sayyid: Sandler et al., 2005). However, in some communities, deafness appears to be autosomal dominant (Ban Khor: Nonaka, 2009). In Providence Island, the prevalent form of deafness is both autosomal dominant and syndromic (Washabaugh, 1979).

Unlike national signing communities, such as the ASL-signing community, that predominantly consist of deaf signers, village signing communities may have more hearing members than deaf ones. Consistent with the title of her book (*Everyone Here Spoke Sign Language*), Groce (1985) noted that many hearing residents of Martha’s Vineyard were signers and that they sometimes used MVSL even when no deaf individuals were present. In this way, the Vineyard signing community was like several contemporary village signing communities. Nonaka (2009) reported that 15%–26% of the population of Ban Khor, Thailand (total pop. 2,741) were users of Ban Khor Sign Language. Kisch (2012, p. 369) reported that there were “more than 700 hearing signers” in the Al-Sayyid village, with widely varying levels of fluency in Al-Sayyid Bedouin Sign Language. In Kata Kolok, Bali, some 57% of hearing villagers had at least minimal proficiency in the local sign language (de Vos, 2012b; Marsaja, 2008). Based on a survey of 547 residents of Chicán, Mexico (total pop. 720), Escobedo Delgado (2012, p. 377) reported that there were 121 fluent signers in the village, 211 with “some competence,” and 215 who did not sign. Thus, an estimated 60.7% of that village’s population had at least some competence in Chicán Sign Language, which has also been referred to as Yucatec Maya Sign Language (Le Guen, 2022).

#### The lifespans of village signing communities

There are large differences in the lifespans of village signing communities. The Al-Sayyid Bedouin signing community was roughly 70 years old when Sandler et al. (2005) published their findings. The community in Ban Khor formed in the 1930s (Nonaka, 2009). In the community in which Central Taurus Sign Language emerged, the first deaf individual was born in the 19th century, but Ergin et al. (2018) argue that a sign language emerged more recently, beginning in 1955, in the deaf population’s sixth generation. In Adamorobe, the signing community may have begun to form in the late 18th century (Kusters, 2012); hence, that community has persisted roughly 250 years.

According to Groce (1985), many of the English settlers of Martha’s Vineyard belonged to a signing community that she traced back through Barnstable and Scituate, MA, and further back to communities in the County of Kent, England. On her account and on that of later scholars (Lane et al., 2000; Nash, 2015), the signing community must have formed prior to the 1630s, when settlers from Kent migrated to Massachusetts. If so, this signing community persisted more than 300 years until the mid-20th century.

Evidently, the lifespans of some village signing communities may last centuries. Nevertheless, when compared to speech communities, village signing communities and their languages are likely to be short-lived. Why? The population may cease to be reproductively isolated. Improved transportation may bring migrants to the community who intermarry with the resident population, but who do not carry the gene for deafness, thereby leading to fewer deaf births (Groce, 1985). Deaf members of the community may be induced to migrate because of educational and employment opportunities elsewhere (Ergin et al., 2018; Nonaka, 2014). Increased educational opportunities may bring the national sign language into the village signing community (Cumberbatch, 2012; Kisch, 2012). For these reasons, the signing community may decline in numbers or may merge into the national deaf community, sometimes leaving hearing individuals as the last signers of the village sign language (Lanesman & Meir, 2012).

### Our goals

Using demographic, geographic, and genealogical data, we will trace the emergence, growth, and decline of the deaf population on Martha’s Vineyard. We will present a variety of analyses, including (i) the deaf population’s size and structure over time; (ii) the education of deaf islanders at schools for the deaf; (iii) the ages of deaf islanders at marriage and whether they married deaf or hearing individuals; (iv) the deaf population’s fertility and mortality; (v) its geographic distribution across the island’s communities; and (vi) estimates of the size of the core signing community on the island, including deaf-parented hearing individuals, as well as the hearing siblings and spouses of deaf islanders. We will use these data to identify historical periods in the lifespan of the Vineyard signing community. Linguistic data on MVSL will not be considered in this paper, except in passing; see Orfila (2024) for a detailed treatment of the linguistic data. Nonetheless, the demographic analyses will have important implications for our understanding of the emergence and death of MVSL—in particular, the effects of contact with emergent ASL beginning in 1825.

Throughout this paper, we will distinguish between the genetic population that apparently hosted the recessive gene for deafness and the signing community that arose among deaf and hearing individuals on Martha’s Vineyard. This distinction will have important implications for our understanding both of the size of the signing community and of when and where that community arose on the island. Analyses of the education of deaf islanders and of their marriage patterns will allow insights into the social dynamics of that signing community. We also draw on oral history interviews with hearing islanders from the 1970s onward (e.g., Bahan & Nash, 1995; Groce, 1985; Lee, 1998). We will track the lifespan of the Vineyard signing community from the *founding* of the island’s deaf population in the 1690s through the apparent *spread* of the recessive gene, particularly within the communities of Chilmark and Tisbury, followed by the growth of the deaf population and the *flourishing* of a signing community in Chilmark and western Tisbury from 1785 to the late 19th century. The deaf population then began a long *decline* through 1998, when the last deaf individual known to be a descendant of island families passed away. The signing community had largely disappeared by the 1950s, but hearing signers lived through the end of the 20th century.

In our conclusion, we address the implications of our work for understanding Lane et al.’s (2000) distinction between assimilating and differentiating societies. Drawing on our demographic analyses and on oral history interviews, we will conclude that the Vineyard signing community reflected aspects of both types of societies. As we will show, the founding of ASD occurred early in the formation of the signing community in Chilmark. Post-1825, deaf islanders quickly integrated themselves into the nascent New England signing community before the Vineyard signing community had had much time to develop independently.

## Sources and methods

A full discussion of our sources and methods can be found in the online supplementary materials. There, we discuss the sources we used to understand the deaf population and the signing community on Martha’s Vineyard; those sources include various documents from the 18th and 19th centuries, census and genealogical records, the records of Alexander Graham Bell’s research into New England families with deaf members, and ASD's enrollment records (Power & Meier, 2023), as well as prior scholarship about Martha’s Vineyard (especially Groce, 1985 and Lane et al., 2011). Although many primary sources are now available online, others can only be accessed in libraries or archives. During this project, we visited ASD's archives in Hartford, CT, the Martha’s Vineyard Museum, the Gallaudet University Archives in Washington, DC, and the archives of the Alexander Graham Bell Association in Chantilly, VA. We also consulted the Alexander Graham Bell Family Papers in the Library of Congress.

In the supplementary Sources and Methods section, we describe the databases of deaf and hearing islanders that we have compiled, as well as our methods for addressing challenges that confront research on the deaf population in the U.S. before 1850. For example, we describe our approach to identifying the deaf individuals who were enumerated in the 1830 and 1840 federal censuses, but who were not identified by name.

## Results

Here, we report analyses of (i) the size and structure of the deaf population on Martha’s Vineyard over time; (ii) deaf islanders’ attendance at schools for the deaf; (iii) their marriage patterns and, relatedly, the migration patterns of mainland-born and island-born deaf individuals, whose moves to the island or away from it were typically linked to marriage; (iv) the fertility rates of the deaf population and the births of deaf children into it; (v) the deaf population’s mortality; and (vi) the approximate locations of deaf islanders’ residences. Finally, we estimate (vii) the size of the Vineyard signing community, including hearing signers.

### Demographic analysis of the Vineyard deaf population, 1692–1998

#### Population size and structure

Between 1692 and 1998, there were 53 known deaf residents of Martha’s Vineyard (29f, 24m). Their mean lifespan was 66.0 years (*SD* = 24.8, median = 76.5). On average, male residents (*M* = 70.0, *SD* = 19.6, median = 76.2) lived 7.3 years longer than females (*M* = 62.7, *SD* = 28.0, median = 76.5). Forty-five deaf island residents (24f, 21m) were born on the island, whereas eight (5f, 3m) were born on the U.S. mainland: five in Massachusetts and one each in Maine, Connecticut, and Rhode Island.

##### Who was deaf and why?

Among the deaf residents of Martha’s Vineyard, 48 (26f, 22m) were either identified in historical records as “deaf and dumb” in 19th century parlance—that is, “deaf” in today’s language—or they attended a school for the deaf; four (3f, 1m) were identified as “deaf” (i.e., hard-of-hearing), either by Bell or in contemporaneous sources; and one male was identified by Bell as “mute.”

For many of these individuals, we have no information about the causes of their deafness. However, for those who attended ASD (see the Education section below), ASD’s enrollment records list the causes of their deafness for each student when known; presumably, that information was based on the reports of those individuals who accompanied the deaf students to Hartford for their enrollment. Of the 25 island-born students who attended ASD, 21 are listed as having been “born deaf” or “congenital.” No cause of deafness is given in Alfred Mayhew’s enrollment record, but, like his four deaf siblings, he was almost certainly congenitally deaf. One student (Caroline Hammett) was said to have been “born partially deaf”; Caroline’s sister, Mary Olive, did not attend ASD, but she was referred to as “deaf,” not “deaf and dumb,” by Bell. Thus, both sisters may have had some hearing and may have used speech. Only two island-born students are thought to have become deaf after birth. Henry Luce’s cause of deafness is given as “unknown” in ASD’s records. His son reported that he was not born deaf (Luce, 1984), and he was not identified as deaf in the 1880 federal census, taken in June of that year, when he was nearly 4 years old. Prudence Lambert is reported to have become deaf after birth. Her record states: “[D]eafness caused by inflammation in the head at 7 months old.”

Of the five deaf island residents born off-island who attended ASD, three (Hannah Smith, Harriet Closson, and Sabrina Rogers) are said to have been born deaf. For one woman (Sarah Foster), ASD lists the cause of deafness as “unknown,” but she had two deaf siblings and hence she is likely to have been genetically deaf. Catharine Dolan, who according to ASD became deaf at age 2, is reported to have retained some speech into adulthood (Huntington, 1983).

In sum, for those 30 deaf islanders whose cause of deafness is known, or readily inferable, 27 (16f, 11m) were born deaf or hard-of-hearing, and three became deaf after birth.

##### Length of residence

Deaf individuals resided an average of 54.0 years on the Vineyard (*SD* = 25.6, min = 3.4, max = 97.5, median = 53.9 years). Just one known deaf individual lived for fewer than 10 years on the island; Mary B. Smith died as a young child.^2^ On average, male residents lived approximately 17.4 years longer on the island than did female residents; see Table 1. This disparity can be partly explained by two points: More females than males emigrated off-island (8f, 2m) and three deaf female students died while attending ASD in Hartford; see the Mortality section. Table 1 shows that women born off-island who later immigrated to the Vineyard resided, on average, 8 years longer on the island than did island-born women.

**Table 1:** Mean length of residence for deaf residents of Martha’s Vineyard, by gender and birthplace.

Gender	Island-born				Born off-island				Overall	
	*n*	*M*	*SD*	*Median*	*n*	*M*	*SD*	*Median*	*M*	*SD*
Females	24	44.7	28.1	32.7	5	52.7	7.1	51.3	46.1	25.9
Males	21	65.8	18.9	73.1	3	47.9	30.8	46.8	63.5	21.6

**Figure 2:**
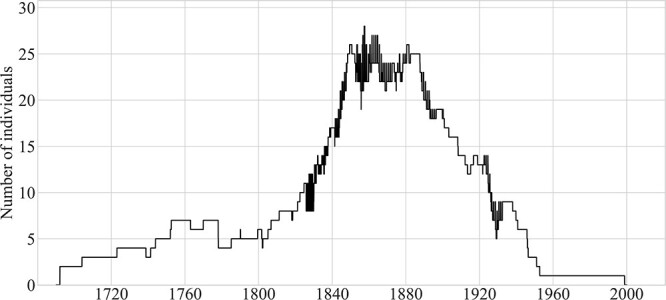
Deaf residents of Martha’s Vineyard, 1690–2000. *Note.* Fluctuations (i.e., dark portions of the line graph from 1825 to 1932) reflect periods during which residents lived off-island for parts of the year while attending schools for the deaf.

Figure 2 tracks the size of the deaf population over time. The figure shows periods of residence on the island. The number of deaf residents sharply increased in the first half of the 19th century, growing from six in 1800 to over 25 by mid-century; there was a brief peak of 28 in mid-1857. The number of deaf islanders was greater than 20 (with only one brief dip below that number) from 1845 until 1893. Beginning in mid-1887, the deaf population declined from 25 individuals to fewer than 10 in 1926 and then to fewer than five from 1946 onward.

##### Mean age of the deaf population

**Figure 3:**
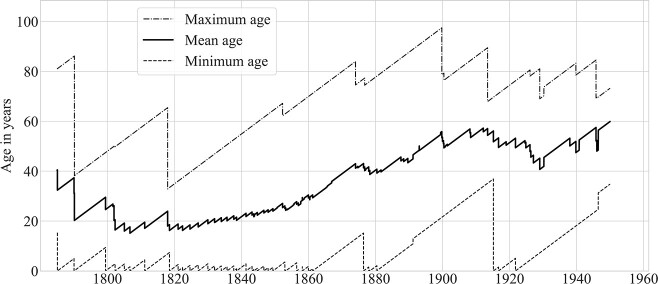
Maximum, mean, and minimum ages of deaf residents of Martha’s Vineyard, 1785–1950. *Note.* Figures calculated by day. The maximum age line up to 30 December 1817 reflects our assumptions about Rebecca Skiff’s age. We assume that she must have died or left Chilmark before the selectmen report, dated December 31, 1817, in which only the Mayhews and Smiths are reported as living in that town; see the online supplementary materials. No other towns on the island submitted reports in 1817; thus, we assume that she was no longer resident on the island at that point. She may of course have left the island at an earlier date.

From the late 18th century through the first half of the 19th century, the deaf population was young. Figure 3 shows that the average age of deaf islanders was below 30 from 1790 until 1859 and under 20 for long periods between 1802 and 1834. The mean age of the population increased over the course of the 19th century, reaching 53.6 in 1900. For comparison, the mean age of the U.S. population was 22.9 in 1900 and 30.2 in 1950 (U.S. Bureau of the Census, 1975).

Comparison of Figures 2 and 3 indicates that the decline in the size of the deaf population that began in 1887 occurred as that population was becoming older on average. The population decline leveled off between 1915 and 1921, with the births of three deaf islanders, before continuing until the end of the 20th century.

#### Education

The advent of deaf education in New England in the early 19th century brought about crucial changes to the lives of the deaf residents of Martha’s Vineyard. Many spent years living in ASD’s dormitories, interacting with deaf individuals from the mainland, learning emergent ASL and written English, and forming relationships that would span decades. This change in the educational opportunities available to deaf islanders led to further changes in their marriage and migration patterns; it also had consequences for their fertility and mortality. Here, we describe the educations of deaf islanders and foreshadow the broader changes described in the sections below on Marriage and Migration, Fertility, and Mortality.

##### Deaf islanders who attended schools for the deaf

On October 25, 1825, Lovey Mayhew and Sally and Mary Smith enrolled at ASD, roughly 8 years after ASD’s establishment in April 1817; it was also in 1825 that Massachusetts extended its funding for education to a large number of deaf students (Power & Meier, 2023). Lovey, Sally, and Mary were the first three deaf islanders to attend a school for the deaf. In total, 28 island-born deaf individuals attended a school for the deaf, whether ASD (25) or the oralist Clarke Institute (four) that was established in 1867; one islander spent time at both schools. The 25 students from Martha’s Vineyard who attended ASD did so for 4.7 years on average (*SD* = 1.5, median = 4.8); the average length of attendance for the four students who attended the Clarke Institute was 5.3 years (*SD* = 3.2). Five deaf women who had attended ASD and who were born off-island moved to the island after their marriages to island-born deaf men. There were thus 30 deaf island residents who attended ASD; they did so between 1825 and 1896. The four students who attended the Clarke Institute did so between 1882 and 1932.

###### Periods of attendance at ASD over time

**Figure 4:**
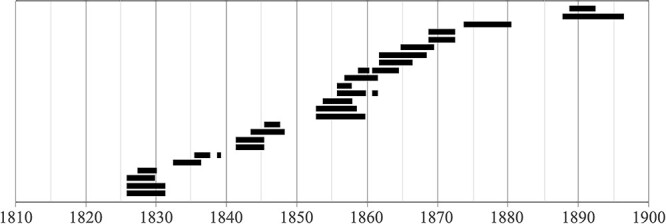
Periods of enrollment at ASD of island-born students from Martha’s Vineyard. *Note.* Each horizontal bar, including discontinuous bars, represents one individual’s period(s) of enrollment.

Figure 4 shows the periods of attendance at ASD of island-born deaf individuals. The highest number of islanders to attend concurrently was six (September 1856–October 1857; see Power & Meier, 2023). Between 1852 and 1872, there was always at least one ASD student from the Vineyard. These students tended to remain at the school for longer periods later in the 19th century. There is a strong, though nonsignificant, positive correlation between their date of enrollment and length of attendance, *r*(23) = .74, *p* = .3, *NS*. This relationship was significant in an analysis of all 1,700 students who attended ASD between 1817 and 1867 (Power & Meier, 2023).

###### Continuing connections to mainland deaf associations

Many deaf islanders remained in contact with their former ASD schoolmates after leaving Hartford. ASD faculty members and former students visited the island in the 1830s and 40s; see Sources and Methods in the online supplementary materials. Deaf islanders also traveled to the mainland. In 1854, three women—Sally (Smith) Mayhew and Ruby and Lovey Mayhew—traveled to Hartford to attend the dedication of a memorial to Thomas Gallaudet. In 1856, 10 deaf islanders, including Mary (Smith) Brown who had moved to Henniker, NH in 1832, were listed among the members of the New England Gallaudet Association (Chamberlain, 1857). They may have attended the Association’s convention in Concord, NH in 1856 (Lane et al., 2011). In 1860, 10 islanders made the trip to Hartford to attend the Association’s fourth convention. In 1866, Benjamin Mayhew (b.1846, son of Benjamin and Hannah Mayhew) attended the 50th anniversary celebration, held in Hartford, of the 1816 arrival in the U.S. of Laurent Clerc and Thomas Gallaudet.

##### Deaf islanders who never attended a school for the deaf

Seven island-born deaf individuals were alive in 1817 or thereafter but never attended any school for the deaf. Why didn’t they? The three siblings Benjamin, Elijah, and Ruby Mayhew may have been too old to enroll at ASD in 1825, when their younger sibling Lovey did so. They were then 40, 35, and 26. In 1825, ASD’s admissions criteria stipulated that students be between ages 10 and 30 (Power & Meier, 2023); although Ruby was eligible to enroll, she did not. Other deaf islanders who never attended a school for the deaf included Frederick Fisher (1860–1924) of Edgartown, as well as Joseph West (1861–1945), Lavinia Smith (1877–1893), and George Trask (1880–1891), all of whom resided in Chilmark. Lavinia may have had multiple disabilities; see her ancestor tree in Box 7 of the Alexander Graham Bell papers at the archives of the Alexander Graham Bell Association for the Deaf and Hard of Hearing, where she is referred to using the now-pejorative term “idiotic deaf-mute.” George died at age 10. Hence, Lavinia may not have been eligible to attend either ASD or the Clarke Institute, and George may not have had sufficient chance to do so—though, at age 8, he would have been eligible to enroll at ASD according to its published policies; see ASD, 1891). It remains unclear why Frederick Fisher and Joseph West never attended a school for the deaf. Joseph’s lack of attendance is curious given that his mother and all four of his older deaf siblings had attended ASD. Lane et al. (2011, p. 100) stated that Joseph West “was reportedly the only illiterate Deaf person in Chilmark.” They did not explain why he may have been illiterate.

In sum, through their attendance at ASD, or through the attendance of a family member, many deaf islanders became integrated into the New England signing community. Many islanders actively maintained their connection to that wider signing community by traveling to gatherings of deaf individuals on the mainland and by becoming members of deaf associations.

#### Marriage and migration

After 1825, many deaf islanders married mainlanders, most of whom were former ASD students. These marriages drove patrilocal migration patterns. Here, we describe the marriages and migration patterns of deaf islanders, which are key to understanding other demographic parameters, such as fertility (see below), that affected the Vineyard deaf population.

##### Marriage patterns among deaf islanders

Roughly one-half of all deaf islanders (32/53, 60.4%) married. Table 2 shows that 40 island-born deaf individuals are known to have lived until at least age 18; five females died before 18. Of these 40 adults, 26 (65%; 12f, 14m) married at least once. Three of these 26 (2f, 1m) married twice; all three second marriages were to deaf spouses. There were thus 29 total marriages involving at least one deaf islander. The marriage rates of island-born males (66.7%) and females (63.2%) were similar. Fourteen individuals (7f, 7m; i.e., 35% of the adult deaf population) lived past 18 but did not marry.

**Table 2:** First marriages of deaf residents of Martha’s Vineyard.

Gender	Island-born					Mainland-born				
	*n*	Age 18+	Married	Spouse		*n*	Age 18+	Married	Spouse	
				Deaf	Island-born				Deaf	Island-born
Female	24	19	12	6	1	5	5	5	5	5
Male	21	21	14	5	1	3	2	1	0	0

“Age 18+” indicates the marriageable age.

Considering only the first marriages of island-born deaf individuals, 50% (6/12) of females and 35.7% (5/14) of males married deaf spouses. If we consider all deaf island residents and both first and second marriages, more female islanders married a deaf spouse than did males (13 vs. 6), and more male islanders had hearing spouses than did females (10 vs. 6). There was just one marriage, whether first or second, between two island-born deaf individuals.

##### Comparison of the mean age at marriage of deaf islanders and their hearing siblings

On average, island-born deaf men first married at age 32.1 (*SD* = 11.1, *n* = 14) and women first married at 24.4 (*SD* = 2.3, *n* = 12). Let’s compare those figures to the average age at marriage of the hearing siblings (*n* = 101; 99 island residents) of deaf island residents; see Sources and Methods in the online supplementary materials. We have located the birth and marriage dates for 69 (36f, 33m) hearing siblings of deaf islanders who were themselves resident on the island for some period; this number includes 10 (5f, 5m) deaf-parented hearing individuals. In addition to these 69, nine hearing siblings never married. For 23 hearing siblings, we were unable to find marriage records; for 2 of these 23, we found the names of their spouse but not their marriage date. On average, the hearing female siblings married at age 23.1 (*SD* = 4.0); the hearing male siblings married at age 28.4 (*SD* = 8.4). Thus, on average, both male and female deaf islanders married later than their hearing siblings.

A two-way analysis of variance was performed to analyze the effect of sex and deafness on the age at marriage. The analysis showed, unsurprisingly, that men (*M* = 29.5, *SD* = 9.4) married at significantly older ages than women (*M* = 23.4, *SD* = 3.7), *F*(1, 94) = 16.08, *p* < .001. Hearing status (deaf vs. hearing) was nonsignificant, *F*(1, 94) = 2.35, *p* = .13, *NS*. The interaction effect was also nonsignificant, *F*(1, 94) = .53, *p* = .47, *NS*.

Part of the explanation for the tendency toward later marriage of deaf islanders (*M* = 28.5, *SD* = 9.1) than of their hearing siblings (*M* = 25.6, *SD* = 7.0) may be that deaf islanders attended schools for the deaf, often into late adolescence and early adulthood (Power & Meier, 2023). Excluding three females who died at ASD (see below), deaf islanders graduated from ASD on average at age 19.6 (*SD* = 3.4), with males (*n* = 12, *M* = 19.7, *SD* = 2.3, range = 16.5–25.0) and females (*n* = 10, *M* = 19.4, *SD* = 4.3, range = 14.2–29.0) graduating at similar ages. Considering only island-born ASD students who later married (8f, 9m), there is a weak, nonsignificant positive correlation between these students’ graduation and marriage ages, *r*(15) = .44, *p* = .08, *NS*.

##### Migration patterns

Of the 45 island-born deaf individuals, 10 (8f, 2m) eventually moved off-island; we do not consider school attendance off-island to have been emigration in this analysis. As noted, eight deaf mainlanders (5f, 3m) immigrated to the island. Here, we focus on the migrations of island- and mainland-born deaf women, which likely had important consequences for the deaf population as a whole; see the Fertility section below.

###### Out-migration of island deaf women

Eight island-born deaf women emigrated to the mainland: one in the 18th century, five in the 19th century, and two in the 20th. Seven women emigrated after their marriages to mainland-born spouses. Six of these women’s spouses were deaf, and one spouse was hearing. Five of the six deaf spouses were former ASD students.

Who were these women? Jerusha Mayhew moved to Massachusetts around 1777; she never married. Mary Smith moved to Henniker, NH after her 1832 marriage to Thomas Brown, an ASD alumnus (Lane et al., 2000). Almira Luce moved to Middleborough, MA after marrying a former ASD student in 1855. Her sister Reliance moved to Essex, MA at some point between 1860 and 1865 after her 1859 marriage to a hearing man; she later married a former ASD student. Prudence Lambert moved to Lawrence, MA in the 1860s after marrying a former schoolmate from ASD. Rebecca West also married a former ASD schoolmate in the 1860s and moved off-island to Deerfield, MA. Her sister, Deidamia J. West, moved to Maine in 1900 at the age of 44 after her second marriage to a former ASD student; her first marriage was to Freeman Smith, a deaf islander and former ASD student. One female islander married a deaf man from the mainland in the mid-20th century.

###### In-migration of mainland deaf women

The departures of island-born deaf women were partly offset by the immigration of deaf women from the mainland. Five moved to Martha’s Vineyard after marrying deaf islanders; all five women were former ASD students. Hannah Smith of New Sharon, ME moved to Chilmark after her 1843 marriage to Benjamin Mayhew. Hannah’s hearing parents had emigrated around 1793 from Martha’s Vineyard to Farmington, ME (and later to New Sharon); thus, she and Benjamin likely shared the same recessive gene for Vineyard deafness (Lane et al., 2011). Sarah Foster (Seekonk, MA) also moved to Chilmark in the 1840s; Harriet Closson (Lyme, CT) did so in the 1850s. The two women married the Tilton brothers, Franklin and Zeno. Sabrina Rogers (East Brewster, MA) moved to Chilmark in the 1870s after marrying George West. Finally, Catharine Dolan (Providence, RI) moved there in 1900 after her marriage to Benjamin West.

##### Summary: marriage and migration patterns

In the 19th and 20th centuries, seven island-born deaf women left Martha’s Vineyard after marriage; at least six were likely carriers of Vineyard deafness. During the same period, five mainland-born deaf women moved to the Vineyard after their marriages to deaf men; these women are less likely to have been carriers. Thus, the marriage and migration patterns of deaf Vineyarders likely reduced the proportion of homozygous carriers of Vineyard deafness within the island’s deaf population. As noted, 35% of island-born deaf adults (14/40) did not marry.

#### Fertility

We now consider the birth rate in the deaf population and the number of deaf children born to deaf–deaf, deaf–hearing, and hearing–hearing couples. We also consider whether these couples likely included carriers of Vineyard deafness. We assume that islanders may have been carriers, while mainlanders likely were not, with one exception that we explain below.

Because Vineyard deafness is thought to be an autosomal recessive trait (Groce, 1985), deaf–deaf couples in which both spouses are homozygous carriers would be expected to have only deaf children. Deaf–hearing couples, with one homozygous and one heterozygous carrier, would be expected to have two deaf births in four. Hearing–hearing couples in which both parents are heterozygous carriers would be expected to have one deaf birth in four. Any marriage between a carrier and a noncarrier of Vineyard deafness would not be expected to result in any deaf births. In the section *The Origins of Vineyard Deafness* in the supplementary materials, we describe a likely source of Vineyard deafness, in particular a mutation to the connexin 26 gene (see Nance, 2004) that is widespread among European populations (Aboagye et al., 2023).

##### Birth rate in the deaf population

The birth rate in the deaf population was relatively low. Fifty deaf island residents were born before 1900.^3^ These deaf islanders had 71 children, or 1.42 children per deaf islander; these figures include children who lived on the island and those who lived off-island. Of these 71 children, 13 were deaf and 58 hearing. The 28 deaf women born before 1900 had 39 children (11 deaf)—a birth rate of 1.4 (.4 deaf), which is lower than the replacement fertility rate of 2.1 in modern societies with low levels of mortality (Lee, 2003).

##### Births of deaf children

**Figure 5:**
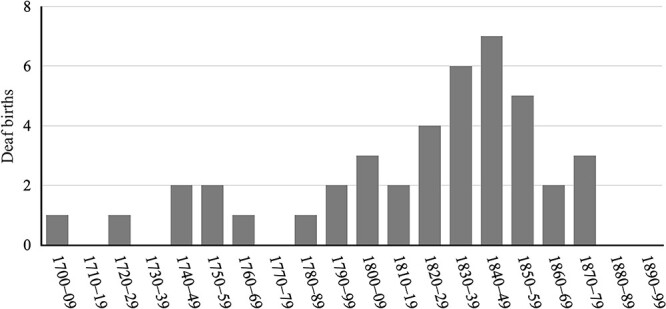
Deaf births on Martha’s Vineyard by decade, 1700–1899.

The number of deaf births was highest in the first half of the 19th century; that number peaked at 7 in the 1840s, as shown in Figure 5. There were 22 deaf births between 1821 and 1859. Deaf births declined in the second half of the 19th century: The first extended period with no deaf births ran from 1861 to 1876. There were again no deaf births from mid-1878 to 1915.

###### Births of deaf children to deaf–deaf couples

Thirteen island-born deaf individuals (8f, 5m) married a deaf spouse. However, just one union included two island-born spouses; Freeman Smith and Deidamia West had one deaf daughter and no other children. As noted, the marriages of island- and mainland-born deaf spouses were unlikely to result in deaf children. Table 3 shows that 5 out of 21 children born to these couples were deaf.

**Table 3:** Offspring of island-born deaf and mainland deaf partners.

Marriage	Island-born spouse	Mainland spouse	Total children	Deaf children	
				Mainland-born	Island-born
1832	Mary Smith	Thomas Brown	2	1	0
1843	Benjamin Mayhew	Hannah Smith	2	0	2
1847	Franklin Tilton	Sarah Foster	3	0	0
1853	Zeno Tilton	Harriet Closson	1	0	0
1855	Almira Luce	Avery Clark	1	0	0
1863	Prudence Lambert	Benjamin Brown	5	0	0
1866	Rebecca West	Eugene Trask	3	1	0
1874	George West	Sabrina Rogers	3	0	1
1900	Benjamin West	Catharine Dolan^a^	1	0	0
1900	Deidamia West	Nathan Pond	0	0	0

^a^According to ASD’s records, Catharine Dolan became “deaf at 2 years old.”

If deaf mainlanders were not carriers of Vineyard deafness, why did some of these couples have deaf children? On the mainland, Thomas Brown and Mary Smith had one deaf son. Thomas is thought to have been a carrier of a dominant gene for deafness (Lane et al., 2000); if so, the fact that Mary was likely homozygous for recessive deafness was irrelevant to whether the couple would have deaf children. Rebecca West and Eugene Trask had one deaf son and two hearing children. Eugene was born deaf and had two deaf siblings; perhaps he was also a carrier of an autosomal dominant form of deafness. On the island, Benjamin Mayhew and Hannah Smith had two deaf children. As a descendant of an island family, Hannah is likely to have been homozygous for Vineyard deafness; thus, it is unsurprising that both children born to this couple were deaf. George West and Sabrina Rogers had one deaf child, Eva West. Sabrina was born deaf and had one hearing sibling; the evidence is too thin for us to speculate about the genetic basis of Sabrina’s deafness.^4^

In sum, deafness in three children in Table 3 can be explained by Thomas Brown’s probable dominant gene for deafness and by the fact that Benjamin Mayhew and Hannah Smith likely were both carriers of Vineyard deafness. Benjamin and Hannah’s homozygosity for that trait meant that all their children would likewise inherit Vineyard deafness.

###### Births of deaf children to deaf–hearing couples

Several deaf islanders married hearing individuals; see Table 4. Some of these hearing spouses are likely to have been heterozygous carriers of Vineyard deafness. At least two hearing spouses in the table were born on the mainland and hence are less likely to have been carriers. The table shows that just two marriages between deaf islanders and their hearing spouses resulted in deaf births. Overall, 7 out of the 48 children born to these couples were deaf. Most of these deaf children were born into one family—that of Deidamia Tilton and George West.

**Table 4:** Offspring of deaf islanders and their hearing partners.

Deaf partner	Hearing partner	Total children	Deaf children
Jonathan Lumbert	Elizabeth Eddy	7	2
Wadsworth Mayhew	Elizabeth Gregory	11	0
Rebecca Skiff	Josiah Mayhew	2	0
Elijah Mayhew	Martha Smith	0	0
Sally Smith	Hariph Mayhew	1	0
Deidamia Tilton	George West	8	5
Charles Luce	Hannah Wilbur^a^	2	0
Mary Hammett	Humphrey Hammett	4	0
Benjamin Mayhew	Harriet West	3	0
Jared Mayhew	Jerusha Reed	1	0
Benjamin West	Mary Hathaway	0	0
Joseph West	Giorgianna Black^a^	0	0
Harry Luce	Irena Mayhew	8	0
Eva West	Henry Look	1	0

^a^Born off-island.

To summarize the information in Tables 3 and 4, all deaf-parented deaf children born into the Vineyard signing community after 1785 belonged to two extended families. Benjamin and Jared Mayhew were the sons of Benjamin Mayhew (b.1785) and Hannah Smith (b.1809). George West (b.1817) and Deidamia Tilton (b.1818) had five deaf children (Rebecca, George, Benjamin, Deidamia, and Joseph) and three deaf grandchildren (Lavinia Smith, Eva West, and George Trask); see below.

###### Births of deaf children to hearing–hearing couples

**Figure 6:**
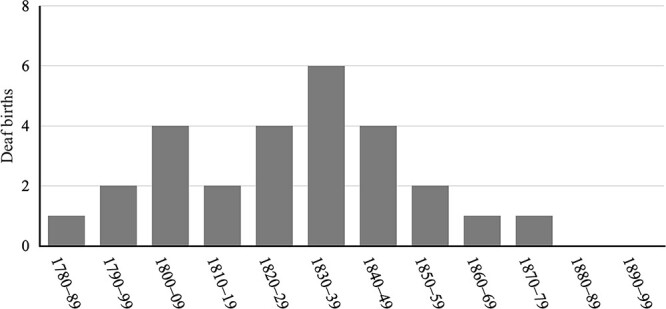
Number of deaf individuals born to hearing couples by decade, 1780–1899.

At the beginning of the sharp increase in the deaf population (see Figure 2), all the deaf children born in Chilmark were born to hearing parents—the Mayhews, Smiths, Tiltons, and Luces. It was not until the 1844 birth of Rebecca West that a deaf child was born to a deaf parent (Deidamia Tilton). Figure 6 shows that 20 deaf children were born to hearing–hearing parents in the first half of the 19th century, but just four were born in the second half of that century. We consider factors affecting the number of births of deaf children to hearing parents in the Discussion section.

#### Mortality

Most deaf islanders had long lives. Table 5 shows that just 11 died before age 40 and 22 lived past age 80. In this section, we report deaf islanders’ known causes of death; we also focus on the tragic deaths of three young women at ASD in Hartford.

**Table 5:** Age at death of deaf residents of Martha’s Vineyard.

Gender	Age deceased						
	<40	>40	>50	>60	>70	>80	>90
Female	8	21	21	19	17	11	1
Male	3	21	20	19	15	11	0

##### Causes of death

For deaf islanders who died in the 18th and early 19th centuries, information about their cause of death is sparse. We have no such information about the first eight deaf individuals who lived on the Vineyard. For 33 of the other 45 deaf islanders, we have found records that include their cause of death. Here we highlight selected recurrent causes. Eight death records indicate “old age” or “senility” as a primary cause of death; some records also indicate an immediate cause, such as “heart disease” or “bronchitis.” Five individuals died of pneumonia (or “lung fever,” as pneumonia was known in the 19th century); three of these five were over the age of 60 when they died.

We have records indicating the cause of death for 8 of the 11 islanders who died before age 40. In 1801, George Pease drowned in a shipping accident in the English Channel (Pease, 1905, p. 252). Jonathan Mayhew died in 1877 of “fits of insanity.” One woman, Rebecca (West) Trask, may have died due to complications following childbirth; although her death record indicates an “unknown” cause of death, she died 9 days after the 1880 birth of her son, George Trask. George died 10 years later of diphtheria. Lavinia Smith died in 1893 of tuberculosis (or “consumption”). In addition to these five individuals, three young women died while at ASD.

##### Deaths of deaf women at ASD

One catastrophic set of events affected the Vineyard deaf population: Three young deaf women died within a short span of time at ASD in Hartford. Catharine Luce and Caroline Hammett died of pneumonia (or “lung fever”) in October and November of 1857 at ages 14 and 16, respectively. Mercy Mayhew died of typhoid fever in 1859 at age 21. Presumably, these women were homozygous carriers of Vineyard deafness. Their deaths considerably reduced the number of women who were most likely to bear deaf children. In 1855, shortly before their deaths, there were 12 island-born deaf women residing on the island; the deaths of these three women reduced this population by 25%.

#### Geography of deafness on the island

In this section, we plot the approximate residences of deaf islanders. We focus on four periods, based on the deaf population curve in Figure 2, during which (i) the population was small and dispersed; (ii) began to grow in Chilmark and (iii) increased sharply there and in western Tisbury; and (iv) declined and again became dispersed.

##### The small and dispersed deaf population

As we have seen, the deaf population on Martha’s Vineyard did not exceed seven individuals until the early 19th century. Before then, deaf islanders were dispersed across the island. Although the island’s three main towns were close (ca. 8 miles from Edgartown to Holmes Hole, part of Tisbury; 12 from Holmes Hole to Chilmark; and 14 from Chilmark to Edgartown), travel between towns was evidently difficult and rare during this period. Groce (1985) reported that, before the advent of the automobile, travel from Chilmark and West Tisbury to Edgartown required a full day over difficult roads; and that even the 5-mile trip between Chilmark and West Tisbury was rarely undertaken.

**Figure 7:**
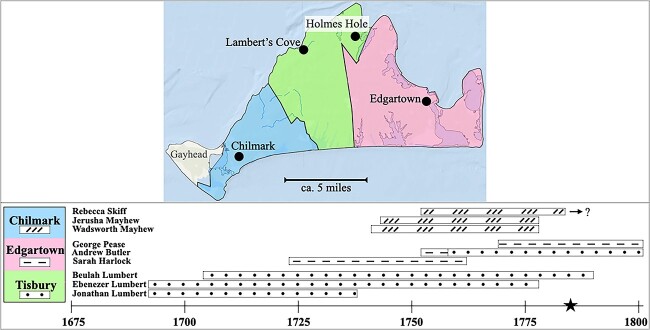
Approximate locations and periods of residence of the nine deaf islanders alive before 1785. *Note.* Each line represents the island residence of one individual. Andrew Butler was born in Edgartown but moved to Holmes Hole in 1758. Rebecca Skiff’s death date is unknown; she was evidently not resident in Chilmark in 1785. ★ = birth of Benjamin Mayhew in 1785.

Nine deaf individuals resided on Martha’s Vineyard before Benjamin Mayhew’s 1785 birth in Chilmark. Recall that this count includes the siblings Wadsworth and Jerusha Mayhew, who were identified as “deaf” in early sources, and Andrew Butler, who was identified as “mute” (see Sources in the supplementary materials). Figure 7 shows the approximate periods of residence of these nine individuals and the parts of the island in which they lived.

The earliest group of deaf individuals settled in Tisbury. The Lumbert family lived near the area in the northern part of the island now known as Lambert’s Cove (Banks, 1911b, p. 53). Ebenezer Lumbert immigrated to the island with his parents at a young age (approx. 4 years old). It is unclear when Ebenezer died; he is named in his father’s 1738 will. Figure 7 (and other analyses in this paper) arbitrarily assumes that Ebenezer lived until age 90. Given this assumption, Ebenezer died before Benjamin Mayhew’s birth. Beulah Lumbert, Ebenezer’s sister, died in 1790, when Benjamin Mayhew was 5 years old. In our view, it is unlikely that the two would have interacted, given the differences in age and residence.

In Edgartown, Sarah Harlock and Andrew Butler may have overlapped for roughly 5 or 6 years. Andrew was born in 1752, but, according to Banks (1925, p. 56), his father (and, presumably, Andrew) moved to Holmes Hole in 1758, when Sarah was roughly 35 years old and Andrew was 5 or 6. Thus, it seems unlikely that the two would have interacted much in Edgartown. George Pease was born in Edgartown in 1769, well after Andrew had moved to Holmes Hole in 1758 and after Sarah’s death in 1762. Both Andrew Butler and George Pease died in 1801. Given the distances between Holmes Hole, Edgartown, and Chilmark, it is unlikely that these men interacted with Benjamin Mayhew.

In Chilmark, the siblings Wadsworth and Jerusha Mayhew were born in 1741 and 1743, respectively. Wadsworth emigrated from the island in 1777 and died in Cambridge, MA in 1829. Jerusha died in 1823 in Conway, MA (Banks, 1925, p. 319). We assume that Jerusha, who apparently never married, emigrated with her brother in 1777.^5^ Rebecca Skiff, who also lived in Chilmark, was born in 1752. Thus, she was about 10 years younger than Wadsworth and Jerusha Mayhew, and she may have concurrently resided in Chilmark with them for roughly 25 years. As we have seen, according to the interviews in Turner (1847), there were no deaf individuals residing in Chilmark in 1785 when Benjamin Mayhew was born. It is unclear where Rebecca Skiff resided then and whether and when she might have moved away from Chilmark. We have been unable to locate any genealogical records for Rebecca Skiff or her husband, Josiah Mayhew, from the time after their 1779 marriage in Chilmark.

In summary, prior to the sharp increase in the deaf population in Chilmark, there were at least two groups of deaf individuals who resided on Martha’s Vineyard. These groups were (i) the Lumbert family who lived in Tisbury near Lambert’s Cove and (ii) the Mayhew siblings (Wadsworth and Jerusha) in Chilmark. Rebecca Skiff apparently resided in Chilmark at the same time as the Mayhew siblings; hence, she may have interacted with the Mayhews and she may have belonged to this group. However, because Wadsworth and Jerusha were both identified by Bell as “deaf,” not “deaf and dumb,” we do not know whether they were signers. Although three deaf individuals resided in Edgartown, they did not do so concurrently as adults.

##### The growth of the deaf population concentrated in Chilmark

**Figure 8:**
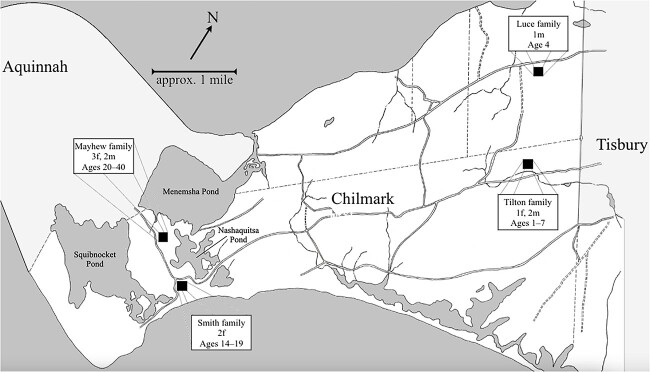
Approximate residences in October 1825 of four Chilmark families with deaf members. *Note.* This map (and the map in Figure 9) is based on Walling’s (1858) map, on annotations of residences by Richard Pease, and on census records.

The growth of the deaf population in Chilmark began in the family of Benjamin (b.1744) and Lydia (b.1764) Mayhew. After their marriage in 1784, the Mayhews resided in a relatively remote part of western Chilmark, in the strip of land between the Menemsha and Squibnocket Ponds; see Figure 8. As we have seen, Lydia Mayhew reported that she met a “deaf and dumb” woman in late 1784 while attending a funeral; and that she “had never seen a person in [the woman’s] condition before” (Turner, 1847, p. 28).^6^ Thus, Benjamin Mayhew’s birth in February 1785 evidently marked the beginning of a new deaf population in Chilmark. In the 20 years following his birth, the Mayhews had four other deaf children—the last of whom, Alfred, was born in 1805. Roughly one-and-a-half years later in October 1806, the Mayhews’ neighbors, Mayhew (b.1779) and Sarah Smith (b.1781), had a deaf daughter named Sally. Sally’s deaf sister, Mary, was born in February 1811. Figure 8 shows that the Mayhews’ and Smiths’ residences, with seven total deaf individuals, were separated by less than 1 mile. The figure also plots the residences of the Tilton and Luce families in eastern Chilmark.

Figure 8 focuses on the period from 1785 to 1825; as noted, the first three deaf islanders enrolled at ASD in Hartford in 1825. In our view, this roughly 40-year period represented the time during which a distinct island signing community may have developed among the deaf and hearing populations in Chilmark, before members of that community encountered the early New England signing community in Hartford.

After 1811, no other deaf individuals were born into the Mayhew and Smith families in western Chilmark. In eastern Chilmark, four deaf children were born to the Tilton (3) and Luce (1) families prior to 1825; another deaf child was born to the Luces in 1827. These Tilton and Luce families resided roughly 5–6 miles from the Mayhew and Smith families. According to the interviews in Turner (1847), Rebecca Tilton (b.1793) had met the Mayhews and Smiths before the 1818 birth of her first deaf child, Deidamia. Thus, the families were in contact. Deaf individuals from all four families would eventually attend ASD (Mayhew = 2/5 children attended ASD; Smith = 2/2; Tilton = 3/3; Luce = 2/2).

##### The growth and spread of the deaf population in Chilmark and Tisbury

As we have seen, there was a sharp increase in the deaf population in the 1830s and 40s. This growth was due to two factors: (i) the births of deaf children in Chilmark (7) and the western part of Tisbury (6), and (ii) the immigration to Chilmark of two former ASD students from the mainland. Inspection of 19th century census records confirms that deaf island residents were concentrated in the western part of the island; see Table 6.

**Table 6:** Deaf residents of Martha’s Vineyard reported in 19th century census records by town.

Census year^a^	Edgartown	Tisbury	Chilmark
1830	0	0	12
1840	0	5	11
1850	0	5	17
1860	0	1	18
1870	1	1	18
1880	2	1	20

^a^Beginning in 1870, the populations of Gayhead and Gosnold were reported separately. In 1880, the population of Cottage City was reported separately. No deaf residents were reported in these towns.

**Figure 9:**
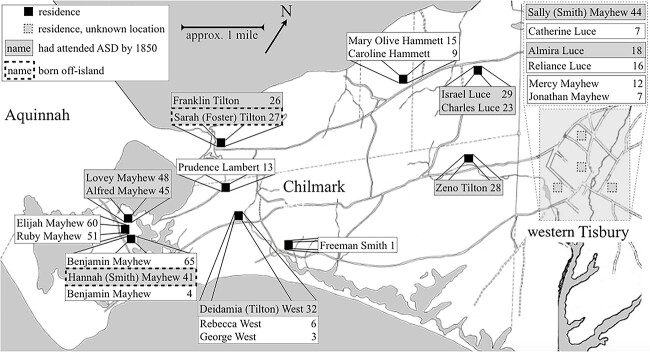
Deaf residents of Chilmark and western Tisbury in 1850. *Note.* Map annotations include the ages of deaf individuals, whether they had attended ASD in Hartford, and whether they were born on- or off-island. Locations of residences in western Tisbury are unknown; we do know that deaf individuals lived in four households. Map of western Tisbury based on Walker’s (1891) map.

Figure 9 shows the approximate residences of deaf individuals in 1850. We focus on 1850 because it falls within the peak period of the island’s deaf population and because Pease’s annotations of the map of Chilmark occurred around that time (see the Sources in the supplementary materials). Although the 1850 census counted 17 deaf individuals in Chilmark, Figure 9 shows that there were then 19 deaf islanders residing there. (Mary Olive Hammett was likely hard-of-hearing in contemporary parlance and was not identified as deaf in the census. Freeman Smith was 1 year old and was also not identified as deaf.) Whereas there were no deaf individuals residing in western Tisbury in 1825 (see Figure 8), by 1850, there were six.

Deaf individuals sometimes moved between and within island towns in the western part of the island. Sally (Smith) Mayhew, who had resided in Chilmark in 1825, moved to western Tisbury after marrying Hariph Mayhew in 1832. At that time, there were no deaf adults residing in Tisbury. Deidamia (Tilton) West moved from the eastern part of Chilmark to the town center after her 1842 marriage to George West (hearing, b.1817 in Chilmark).

##### The declining and dispersed deaf population

The island’s deaf population remained above 20 until 1893 and above 19 until 1900 (see Figure 2); during that time, deaf residents continued to be concentrated in the western part of the island. However, the population was aging (see Figure 3). Several of the deaf individuals born in Chilmark in the early part of the period (1785–1825; see Figure 8) died between 1880 and 1910. The five Mayhew siblings had all died by 1899. Deidamia (Tilton) West and her brother Zeno Tilton died in the 1880s. The two Luce brothers, Isaac and Charles, died in the early 1900s. Table 7 shows the deaf population by town between 1900 and 1960.

**Table 7:** The deaf population on Martha’s Vineyard in the 20th century, by island region.

Decade	Chilmark		Tisbury and West Tisbury	Other towns
	Island-born	Born off-island		
1900	10	3	1	2
1910	8	2	1	2
1920	6	2	2	3
1930	2	1	2	4
1940	2	1	0	4
1950	1	1	0	1
1960	0	0	0	1

From 1900 to the 1920s, a sizeable group of deaf individuals (8–13) resided in Chilmark, but the population in this western part of the island was nonetheless declining. One mainland-born deaf individual and four island-born deaf individuals died in the 1920s. From the 1930s on, the deaf population was small and dispersed. The table shows that all the 20th century deaf residents of Martha’s Vineyard who were born off-island resided in Chilmark.

#### A final overview of the deaf population

In sum, the deaf population on Martha’s Vineyard was relatively small (fewer than seven individuals) until the early 19th century. Between 1811 and 1845, the deaf population in Chilmark and western Tisbury grew from 7 to 25 and remained above 20 individuals for nearly a half-century (1845–1893), peaking at 28 in 1857. In 1825, during the period of rapid growth, islanders began attending ASD; 25 island-born individuals attended the school between 1825 and 1896. This change in educational opportunities led to changes in marriage patterns, migration, and, likely, fertility. The population began to decline in the 1880s due to a combination of factors, including the deaths of three female islanders in the 1850s, the emigration of female islanders to the mainland for marriage in the 1850s and 60s, and the migration to the island of off-island deaf women who, except for Hannah Smith and, possibly, Sabrina Rogers, are not likely to have shared the same genetic basis for deafness as was typical on the Vineyard. Fourteen adult islanders did not marry. There were no births of deaf children on the island in the 1880s or 90s. We discuss additional factors affecting both the deaf and hearing populations in the Discussion section below.

### Estimating the size of the hearing signing population on Martha’s Vineyard

We estimate the number of hearing signers in two ways: (i) by applying figures drawn from the literature on extant village sign languages and (ii) by determining the number of hearing family members who were likely to have been users of MVSL.

#### Estimates based on the literature on modern village sign languages

Deaf residents of Martha’s Vineyard formed only part of the signing community; many hearing residents were also signers (Groce, 1985). Can we estimate their numbers? One way is to use published estimates of the number of hearing signers in contemporary village signing communities—such as in Ban Khor, Thailand, in Al-Sayyid, Israel, in Kata Kolok, Indonesia, and in Chicán, Mexico—to derive a rough estimate of the number of hearing signers on Martha’s Vineyard in 1850, when the deaf population was at its peak (see Figure 2). The estimated percentages of the total hearing population who were signers in those communities range from 15% to 61% (see Introduction). Given that the island’s total population was 4,526, this would suggest that there may have been between 679 and 2,761 hearing signers on the Vineyard.

However, the deaf population on Martha’s Vineyard in 1850 was concentrated in Chilmark (19) and western Tisbury (6); there were no deaf residents in Edgartown. If we now assume that hearing signers were more likely to be found in towns where deaf individuals lived and if we consider only Chilmark (pop. 747) and Tisbury (pop. 1,789), then we estimate that there were 380–1,547 hearing signers in those two towns (Chilmark = 112–456; Tisbury = 268–1,091). However, because there were more deaf residents in Chilmark than in Tisbury, we might also expect that there were proportionally more hearing signers in Chilmark. If we base our estimate of the number of each town’s hearing signers on the size of its deaf population, we would expect 3.2 times more hearing signers in Chilmark than in Tisbury. Dividing our previous estimate of the Chilmark signing population by 3.2, we now estimate just 35–143 hearing signers in Tisbury. Thus, using this method, we estimate the number of hearing signers on the island in 1850 to have been 147–599 (out of a total population of 4,526).

#### Hearing family members who were likely signers

Can we derive more principled estimates of the size of the signing community on Martha’s Vineyard? Three groups of hearing individuals were particularly likely to have been signers: (i) the hearing children of deaf islanders, (ii) the younger hearing siblings of deaf islanders, and (iii) the hearing spouses of deaf islanders. Deaf-parented hearing individuals would have been exposed to sign from birth. Although not all such individuals are fluent users of a sign language in adulthood (Chen Pichler et al., 2018; Pizer et al., 2013), we will assume that their fluency ranged from competent to native-like. We will also assume that the younger hearing siblings of deaf islanders would have been exposed to sign from birth, and we assume that the hearing spouses of deaf islanders would have signed regularly. For example, Henry Look, who was the hearing husband of Eva West, a deaf islander, reportedly signed regularly (Groce, 1985). Although other hearing individuals, such as friends and fellow church members, may also have been members of the signing community (Groce, 1985), it is challenging to identify these groups and hence to measure their sizes.

##### Deaf-parented hearing individuals

**Figure 10:**
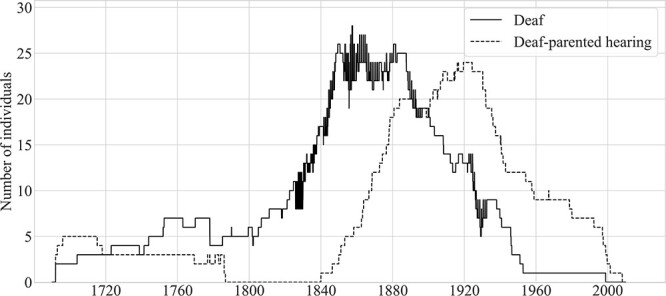
Deaf residents of Martha’s Vineyard and deaf-parented hearing island residents, 1690–2010. *Note.* There was a peak of 24 deaf-parented hearing individuals between 1916 and 1924.

There were 56 deaf-parented hearing individuals whose parents had at some point resided on the island. Only 38 of these 56 individuals themselves resided on the island. The average length of residence of these 38 individuals was 63.1 years (*SD* = 27.4, median = 73.5 years, min = 1.2, max = 93.7). The other 18 deaf-parented hearing individuals were born to deaf individuals who had previously been residents of Martha’s Vineyard but who had moved off-island.^7^ These 18 hearing individuals never resided on the island; hence, we assume that they did not form part of the Martha’s Vineyard signing community. Figure 10 compares the number of deaf island residents to the number of deaf-parented hearing individuals who were residents of Martha’s Vineyard.

Inspection of Figure 10 reveals that the increase and subsequent decline in the population of deaf-parented hearing individuals roughly paralleled that of the deaf population—with the peak in the deaf-parented population lagging the peak period in the deaf population by some 40 years. The total population of deaf-parented hearing residents was lower than the total population of deaf island residents (38 vs. 53); relatedly, the peak number of deaf-parented hearing residents was lower than the peak number of deaf residents (24 vs. 28). From 1717 to 1891, there were at least as many deaf island residents as deaf-parented hearing islanders; for most of that period, the deaf population was much larger. That changed at the end of the 19th century: Between 1891 and 1900, there were roughly even numbers of deaf individuals and deaf-parented hearing individuals; after 1900, there were always more deaf-parented hearing islanders.

We have records documenting the marriages of 36 of the 56 deaf-parented individuals; 13 never married, and we are unsure about 7 individuals. None of the deaf-parented hearing individuals for whom we have marriage records married a deaf spouse.

##### Younger hearing siblings of deaf islanders

At least 101 hearing island residents had a deaf sibling (24 families); 12 of these individuals had at least one deaf parent. There were thus 89 hearing island residents who were not deaf-parented and who had at least one deaf sibling. These 89 individuals were separated from their nearest deaf sibling in age by an average of 6.0 years (*SD* = 4.4, median = 5.0), with 45 individuals who were older than their nearest deaf sibling and 44 who were younger. In addition, seven deaf-parented hearing individuals had at least one older deaf sibling. Thus, there were 51 hearing individuals who had an older deaf sibling.

Let’s consider the 44 hearing individuals who had at least one older deaf sibling and who did not have a deaf parent. They may have been exposed to sign by their older deaf sibling(s) from birth; hence, like deaf-parented individuals, these hearing individuals with older deaf siblings may have been fluent signers. These 44 individuals belonged to 17 families with varying numbers of deaf siblings: Seven families included one deaf sibling, eight families included two, one family included three, and one family included five. On average, the younger hearing siblings of deaf islanders were 5.1 years younger than the deaf sibling closest in age to them (*SD* = 3.1, median = 4.2, min = 1.2, max = 12.9).

**Figure 11:**
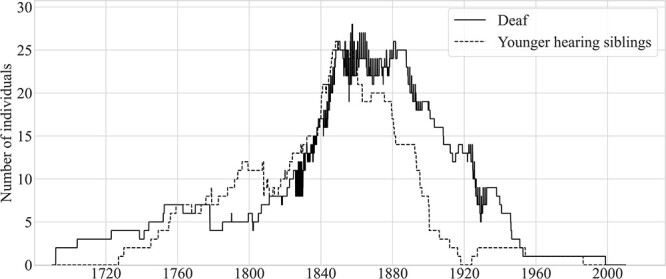
Deaf residents of Martha’s Vineyard and their younger hearing siblings who were resident on the island, 1690–2010.

Figure 11 compares, over time, the number of younger hearing siblings who were resident on the island to the number of deaf island residents; the figure excludes deaf-parented siblings (see Figure 10). Inspection of Figure 11 reveals that, from 1808 to 1860, there were roughly equal numbers of deaf islanders and their younger hearing siblings, with both population curves increasing sharply during that period. The number of younger siblings peaked at 26 in 1848 and 1849. Between 1860 and 1900, that number fell from 24 to just 4. Between 1918 and 1924, there were no younger hearing siblings of deaf islanders living on the island.

The decline in this population preceded the decline in the deaf population by roughly 20–40 years. Why? Many more hearing siblings (14, and possibly an additional 6) emigrated off-island than did deaf islanders (10). Six younger siblings had likely moved to Maine by 1820; four were from Edgartown and two from Chilmark. Another four younger siblings, all from Chilmark and western Tisbury, moved to California between 1849 and 1879.

Who were these younger siblings of deaf islanders? Consider Austin Smith (b.1813), who had two older deaf siblings, Sally (b.1806) and Mary (b.1811). Austin was a captain who, except when at sea, resided in Chilmark. In 1842, he married Lavinia Poole, a hearing woman; the couple later had two deaf children (Freeman and Mary) and two hearing children (Austin and Althea). The elder Austin Smith was the only hearing island resident who had an older deaf sibling and a deaf child. He may have been exposed to sign from birth by his two older deaf sisters, and, as a potentially fluent signer, he may have exposed his two deaf children to sign at an early age. The children were likely also exposed to sign by their deaf aunt, Sally, who resided in western Tisbury; recall that their other deaf aunt, Mary, had moved to New Hampshire in 1832. Thus, although Captain Smith was not deaf, his children had similar backgrounds to those of second-generation signers. One of these children, Freeman, married another deaf islander in 1875. Freeman’s spouse, Deidamia J. West, was herself a second-generation signer, the daughter of Deidamia Tilton (deaf) and George West (hearing). The couple had a deaf daughter, Lavinia Smith. Hence Lavinia was a third-generation signer. Tragically, Lavinia died at age 15 in 1893 (Lane et al., 2011).

Captain Smith’s older deaf sister, Sally, married a hearing man, Hariph Mayhew (b.1791). As noted, the Mayhews and Smiths were close neighbors in the western part of Chilmark (see Figure 8). Hariph had five deaf siblings, including two older deaf brothers, Benjamin (b.1785) and Elijah (b.1790). To our knowledge, Hariph was the only hearing island resident who married a deaf individual and who had a deaf sibling, whether older or younger.^8^

##### Hearing spouses of deaf individuals

In addition to Hariph Mayhew, 14 other hearing island residents married deaf spouses. These 15 hearing spouses (9f, 6m) are likely to have been frequent signers, particularly after marriage. On average, they resided on the island for 41.9 years after marriage (*SD* = 20.5, median = 51.9, range = 2.2–65.0); female and male hearing spouses resided on the island for roughly similar lengths of time after marriage (f: 41.7 years, median = 52.6; m: 42.2 years, median = 50.2). Between 1862 and 1930 (excluding one 8-year period from 1897 to 1905), there were more than five hearing spouses of deaf individuals on the island. Between 1876 and 1880 there was a peak of eight hearing spouses.

#### Summary: size of the hearing signing population

Between 1692 and 2008, there were at least 141 hearing island residents who, by nature of their relationships with deaf islanders, may have been fluent, or at least frequent, signers: These included 38 deaf-parented individuals, 89 siblings of deaf islanders (44 younger siblings), and 14 spouses of deaf islanders. Together with the 53 deaf island residents, these 141 hearing individuals may have formed the core of the signing community, or signing communities, that formed during this roughly 300-year period.

**Figure 12:**
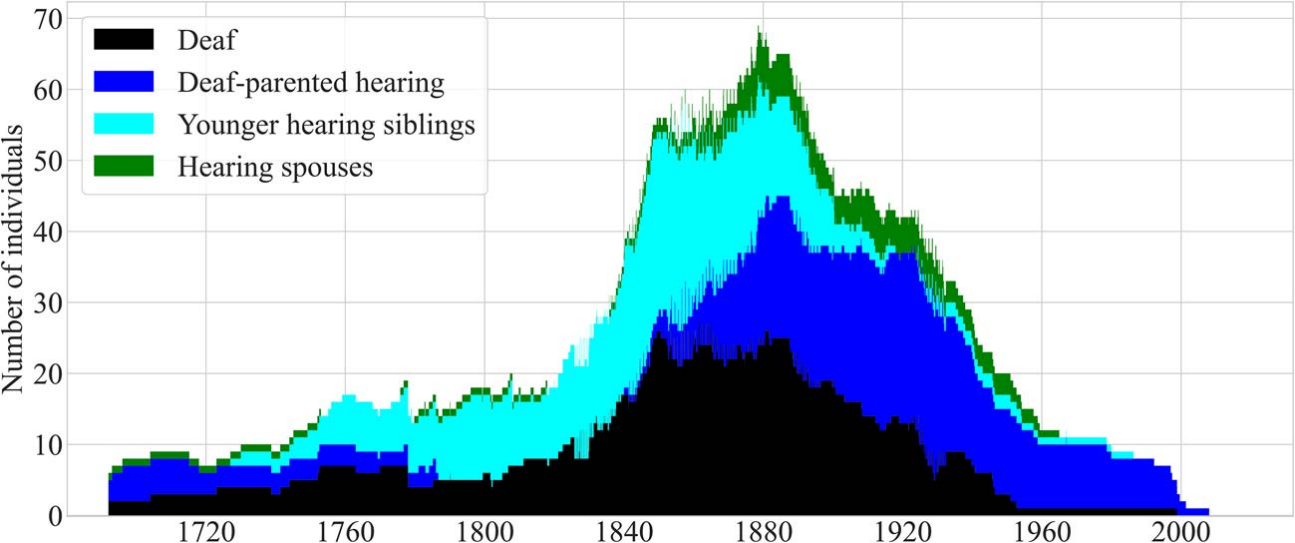
Combined counts over time of four groups likely to have been fluent signers and island residents, 1692–2008.

Figure 12 shows that the population of deaf island residents (25), deaf-parented hearing islanders (18), younger hearing siblings (19), and hearing spouses (7) of deaf islanders peaked at 69 from June to September of 1878. Except for brief periods, these four groups totaled at least 50 individuals from 1846 to 1897 and at least 60 from 1872 to 1889.

Let’s consider again the estimates of the size of the signing community that we based on figures drawn from the literature on village sign languages. We estimated above that the Vineyard community may have comprised 147–599 individuals in 1850. However, we now estimate that there were just 56 members of the core signing community: 26 deaf individuals, three deaf-parented individuals, 25 younger siblings, and two spouses.

As we have seen, the deaf population began to decline in the late 19th and early 20th centuries. By 1926, there were fewer than 10 deaf island residents, and fewer than five by 1946. The population of deaf-parented hearing individuals declined later: There were more than 10 such individuals until 1958 and more than five until near the end of the 20th century. The population of younger hearing siblings had declined much earlier. That group fell below 10 individuals in 1895 and below five in 1900. To our knowledge, no members of this core Vineyard signing community are alive today.

## Discussion

In this section, we describe the lifespan of the Martha’s Vineyard signing community, beginning with the precursory stage that extended from the Lumbert family’s arrival on the island around 1692 until Benjamin Mayhew’s 1785 birth in Chilmark. It was there, in the western part of the island, that the Vineyard signing community began to form and where MVSL emerged. We describe the community’s growth and decline in relation to the nascent New England signing community. Contact with this mainland community—in particular at ASD in Hartford—would contribute to the eventual decline of the island signing community. We also discuss trends that likely contributed to the decline in deaf births to hearing–hearing couples.

Our account of the lifespan of the Vineyard signing community differs from prior accounts of this community in three respects. First, in our view the community formed on the island—that is, it was a novel signing community with no connection to prior signing communities in Kent. Second, the signing community in which MVSL arose formed in the late 18th century in Chilmark, beginning with the births of Benjamin Mayhew in 1785 and his brother Elijah in 1790. Connections to any earlier signers on the island were likely tenuous, given the results of our demographic analyses and given the parental reports in Turner (1847). Third, this signing community, and its language, developed independently of signing communities on the mainland for only 40 years (roughly 1785–1825). After 1825, the Vineyard signing community was quickly integrated into the New England signing community, and ASL likely had a significant impact on MVSL, an impact that strengthened over time. On our account, deaf individuals were continuously present at the core of the Martha’s Vineyard signing community for roughly 165 years, from 1785 to the 1950s; after the 1950s, only hearing signers survived.

### Dispersed deaf population and the spread of a recessive gene for deafness, 1692–1785

Jonathan Lumbert and his two children were the only deaf residents of Martha’s Vineyard early on. Several questions about the Lumbert family remain unresolved; see the sections on Sources and Jonathan Lumbert in the supplementary materials. However, if we assume, as prior scholars have done, that the Lumbert family included three deaf individuals, we can also infer that Jonathan’s hearing wife and children are likely to have been frequent signers; many of the Lumbert family’s neighbors may also have been signers.

What about other deaf island residents during this early period? The deaf members of the Lumbert family were the only deaf islanders for at least 31 years, from the family’s immigration to the island around 1692 until Sarah Harlock’s birth in 1723 (see Figure 7). But Sarah lived in Edgartown—some 8 miles from the Lumberts in Lambert’s Cove at a time when travel between towns was challenging (Groce, 1985). The next deaf islanders were born after the elder Jonathan’s 1738 death. The two Mayhew siblings, Wadsworth and Jerusha, were born in 1741 and 1743 in Chilmark (9 miles from Lambert’s Cove). They were both identified as “deaf,” not “deaf and dumb,” by Bell. Andrew Butler, who was born in Edgartown in 1752 but moved to Holmes Hole in 1758, was identified as “mute” by Bell. It is unclear whether the Mayhews and Butler would have been signers. Rebecca Skiff was born in 1752 in Chilmark, and George Pease was born in 1769 in Edgartown. Both individuals were identified as “deaf and dumb” and hence may have been signers. However, again, both lived a considerable distance from the Lumberts in Tisbury. Hence, the Lumbert family may have been geographically separated from other deaf individuals on the island. Any sign language that arose within that family may have been confined to the area near present-day Lambert’s Cove.

Even if the Lumbert family was geographically separated from other deaf islanders, their sign language may have shared similarities with other signed communication on the island. Goldin-Meadow and Mylander (1998) showed that the homesign systems of children in the U.S. and Taiwan share structural features, such as the order of the arguments within a signed sentence. Neveu (2019) studied a sign language that emerged among three geographically distinct villages in the Peruvian Amazon, in which two deaf individuals (and perhaps a third deaf individual who had died prior to Neveu’s fieldwork) shared sign vocabulary despite rarely interacting directly. Neveu’s explanation for the shared vocabulary among these deaf individuals relies in part on their shared network of hearing interlocutors; see also Reed (2021). On Martha’s Vineyard, we would need to know more about the structure of the island’s social networks and about the frequency of travel between towns in the 18th century to understand whether sign vocabulary could have been widely shared on the island.

In sum, based on our demographic and geographic analyses, and based on the reports of the Chilmark families (Turner, 1847), deafness was infrequent on Martha’s Vineyard in the century separating the Lumbert family’s 1692 arrival in Tisbury and Benjamin Mayhew’s 1785 birth in Chilmark. We do not know whether there was a widespread sign language in use among deaf and hearing islanders, but that seems unlikely. It is perhaps more likely that several distinct homesign systems developed in the families, and perhaps among the neighbors, of deaf individuals in various parts of the island (see Hou, 2016). In our view, these homesign systems were likely distinct from the language that arose in the Martha’s Vineyard signing community beginning in the late 18th century.

The distinction we make between the small, dispersed groups of deaf individuals in this early period and the Vineyard signing community that began in 1785 diverges from prior scholarship that has understood the Vineyard signing community to have begun with the Lumbert family’s arrival on the island (e.g., Groce, 1985; Lane et al., 2000; Nash, 2015). One difference between our analysis and Groce’s lies in the claimed size of the deaf population. We have identified 53 total deaf island residents, whereas she identified “at least 72” (p. 3). We note that her figure is the same as the figure found in Bell’s list of 72 “Deaf-Mutes of Martha’s Vineyard”; see Figure S2 in the supplementary materials. However, Bell’s list is divided into three parts: “Deaf-Mutes of Vineyard Ancestry residing in the Vineyard,” “Deaf-Mutes of Vineyard ancestry (not residing in the Vineyard),” and “Deaf-Mutes who have married Martha’s Vineyard deaf-mutes.” Thus, many of the 72 individuals in Bell’s list never lived on the island; some, such as the Dillingham sisters, apparently did not know any other deaf people before attending ASD; see Distinguishing Between the Genetic Population of Carriers and the Signing Community in the supplementary materials.

Unlike our analysis, Groce (1985, p. 41) reported that the deaf population peaked at 45 in the 1840s. We find that the deaf population briefly peaked at 28 individuals in mid-1857; see Figure 2. This is a substantial difference of 17 individuals. Groce (1983, p. 136) also reported that there were 14 births of deaf children in the 1840s, whereas we count just seven. Unfortunately, Groce’s data are not reported in a way that would allow us to resolve these differences. With a higher peak deaf population, Groce may have understood the growth in that population to have begun earlier than our analyses suggest; hence, she may have also understood there to have been greater continuity between the deaf individuals in the early period before Benjamin Mayhew’s 1785 birth and those who would be born in Chilmark beginning in the late 18th century.

In summary, in the roughly one century (1692–1785) prior to the bourgeoning of the deaf population in the western part of Martha’s Vineyard, deaf island residents were few in number and dispersed across the island. During this period, the recessive gene for Vineyard deafness evidently spread widely among the Chilmark population. In this way, the almost century-long period leading up to the formation of the Vineyard signing community shared similarities with preliminary stages of other well-studied village signing communities. Nyst (2007) reported that a recessive gene (mutation R134W in the connexin 26 gene) likely spread among the early settlers of Adamorobe, Ghana in the late 18th century (see Kusters, 2012). The deaf population in that community later peaked between 34 and 45 individuals.

Similarly, Kisch (2012) reported that a recessive gene for deafness spread among the descendants of one individual named Al-Sayyid in the eponymously named Bedouin town of Al-Sayyid in Israel. Al-Sayyid moved to that area of the Negev Desert around 1800, but his first deaf descendants, the offspring of four consanguineous marriages, were born starting in 1924. This population was reproductively isolated because, as relative late comers to that area, the Al-Sayyid clan was stigmatized by neighboring groups (Kisch, 2012). The Al-Sayyid deaf population grew rapidly after 1924: According to Kisch (p. 369), there have been “134 deaf descendants among all generations of Al-Sayyid.” The village signing community in Bali has a similar history. De Vos (2012c, p. 130) reported that a recessive gene (mutation to DFNB3; Friedman et al., 2000) began to spread “between approximately four and nine generations ago” (i.e., between roughly 1832 and 1932) in a densely populated, geographically small rural area in northern Bali (see Winata et al., 1995). In 2000, there were 47 deaf individuals in a village of 2,186 (Marsaja, 2008, cited in de Vos, 2012c).

In each of these contemporary signing communities, as in the Chilmark community, a recessive gene for deafness spread within a reproductively isolated population. This common preliminary stage established the conditions for a rapid increase in the number of deaf individuals within each community and the subsequent formation of a signing community. The growing frequency of heterozygous individuals in these communities increased the likelihood that homozygous deaf children would be born to phenotypically hearing individuals.

### The Vineyard community prior to contact with mainland deaf individuals, 1785–1825

We have seen that the deaf population on Martha’s Vineyard grew rapidly in the first half of the 19th century, particularly in Chilmark and western Tisbury. This growth began in a relatively remote part of Chilmark, the area called Squibnocket (south and west of the Nashaquitsa Pond), at first only in one family. For 33 years, from Benjamin Mayhew’s birth in 1785 until the 1818 birth of Deidamia Tilton in eastern Chilmark, all seven deaf residents of Chilmark lived in Squibnocket. Four more deaf children were born in eastern Chilmark between 1818 and 1824. Thus, by 1825, before contact with mainland deaf populations at ASD, there may have been a small signing community in Chilmark that included 11 deaf individuals between the ages of 1 and 40. This 40-year period from 1785 to 1825 was apparently the most isolated period in the community’s history. What was happening among the deaf residents of Chilmark at that time?

We get some insight from Benjamin’s sister, Lovey Mayhew (1802–1899), who attended ASD from 1825 to 1831. She wrote a short essay that was published in ASD’s 1830 annual report. In it, she included a few details about her life before 1825; she wrote that her parents “felt very sorry, because, their three sons and two daughters were deaf and dumb, but they could talk with our signs, and have much pleasure” (ASD, 1830, p. 25). Hence, in the period before 1825, a sign language had evidently arisen in the Mayhew family. Perhaps Sally and Mary Smith came to be part of this signing community as young children. Later, the Tiltons and Luces may also have become part of the community.

These four Chilmark families were not completely isolated from events on the mainland. They had likely learned about the new school for the deaf in Hartford even before 1825. The state legislature passed a resolution in June 1817—just 2 months after ASD’s April opening—calling for a census of deaf individuals living in Massachusetts; see Sources and Methods in the supplementary materials. The Mayhews and Smiths had been counted in Chilmark by the end of 1817; hence, the families may have learned at that time about the existence of ASD and the possibility of sending their deaf children there. Lovey wrote this in the essay cited above: “Before I came to the asylum, I knew that the deaf and dumb pupils came to it. I often asked my father if he would permit me to go there, but he had not enough money” (ASD, 1830, p. 25). It is striking that, although before 1825 deaf islanders like Lovey had apparently never met any deaf individuals from the mainland, she nonetheless desired to attend a school for the deaf.

Several schools for the deaf had opened in Europe by 1825, and accounts of their pedagogical methods were available in the U.S. by the late 18th century (Lang, 2007; Valentine, 1993). In 1815, before ASD’s establishment, Alice Cogswell in Hartford apparently knew a form of the two-handed British fingerspelling system (Edwards, 2012, p. 15; Lane, 1984, p. 179). There is evidence that fingerspelling alphabets were widely known in the U.S. in the 1820s. In 1821, Samuel Akerly, the physician at the New York School for the Deaf, published *Elementary Exercises for the Deaf and Dumb*; he included the one-handed fingerspelling alphabet in its first lesson (Akerly, 1821). Edmund Booth (ASD 1828–1832) of Longmeadow, MA overlapped at ASD with the first four students from Martha’s Vineyard. Booth had already learned the one-handed fingerspelling alphabet before he found out about ASD in 1827 (Lang, 2004). Thus, it is possible that knowledge of fingerspelling systems had spread to the Vineyard before the first students from the island left for ASD.

What about the marriage patterns of deaf islanders during this period? Groce (1985) asked whether there was a difference in the marriage rates of deaf islanders before and after the founding of ASD in 1817. She (p. 79) reported that “[o]n the Vineyard 73 percent of the deaf people born before 1817 married; of these only 35 percent married other deaf people.” According to Groce, the marriage rate of deaf islanders (73%) before 1817 was much higher than that of deaf mainlanders in the 19th century (45%, citing statistics in Fay, 1898). The higher marriage rate of deaf islanders versus mainlanders was thought to indicate that deaf individuals were well integrated into island society.

Although ASD was founded in 1817, that year was not central to the shift in the marriage patterns of the Vineyard’s deaf population described in the Marriage and migration section above. Many deaf islanders who were born prior to that year attended ASD and married former schoolmates. Even Benjamin Mayhew (b.1785), who did not attend ASD, married a former ASD student; he was the earliest-born islander to do so. Table 8 compares the marriage patterns of island-born deaf individuals before and after Benjamin’s 1785 birth. Inspection of the table shows that just two individuals born before 1785 married (25%, 2/8); both married hearing spouses. After 1785, 61.5% of island-born deaf individuals (24/39) married, and 72.7% (24/33) of individuals who reached age 18 did so. Considering only first marriages, 45.8% (11/24) included two deaf spouses; counting first and second marriages, 51.9% (14/27) were between two deaf partners. Female islanders (54.5%, 6/11 marriages) more frequently married deaf partners than did male islanders (38.5%, 5/13 marriages). Conversely, male islanders more frequently married hearing partners (m: 61.5%, f: 45.5%).

**Table 8:** Marriage patterns of island-born deaf individuals born before 1785 and later.

Gender	Born before 1785					Born 1785 or later				
	*n*	Married	Unmarried	Spouse		*n*	Married	Unmarried^a^	Spouse	
				Deaf	Hearing				Deaf	Hearing
Female	4	1	3	0	1	20	11	9	6	5
Male	4	1	3	0	1	19	13	6	5	8

*Note*. The number of individuals born before 1785 includes Ebenezer Lumbert, who moved to the island at about age 4. After 1785, George Trask, who may have moved to the island before age 1, is included. Three (2f, 1m) individuals born after 1785 married twice; all second marriages were to off-island-born deaf individuals who were former ASD students.

^a^Five females and one male did not reach marriageable age (18).

Our analyses indicate that the marriage rates of early deaf islanders were lower than previously reported, whether 1785 or 1817 is taken as the transition point for marriage patterns. Fifteen individuals were born on the island before 1817 (including Ebenezer Lumbert, as in Table 8); all reached age 18. Of these 15, six married (40%); two of these marriages (33%, 2/6) were between deaf partners. Thus, contra Groce (1985, p. 79), the marriage rate of deaf people born on the island pre-1817 is lower than the rate reported in Fay (1898, cited by Groce) for some 19th century deaf mainlanders (40% vs. 45%).^9^ The rate of deaf–deaf marriages for those born on the island before 1817 is similar to the rate reported by Groce (33% vs. 35%). However, as noted, there were just six marriages involving at least one deaf partner; and few deaf individuals concurrently resided on the island before 1817. In sum, these data do not indicate that deaf islanders born pre-1817 were better integrated into their island communities than were mainland deaf individuals into their communities.

Post-1817, the percentage of deaf–deaf marriages may have been higher on the mainland than on the Vineyard. Fay (1898) reported that 72.5%–80% of a sample of 4,471 marriages included two deaf spouses.^10^ In comparison, just 52.2% (12/23) of the marriages that included at least one deaf individual born on the island after 1817 united two deaf partners. Conversely, 47.8% of such marriages included one deaf and one hearing spouse, compared to the mainland range of 20%–27.5% (Fay, 1898). Thus, Groce’s (1985, p. 79) conclusion about the higher frequency of deaf–hearing marriages on the island versus the mainland seems correct.

### Intensive contact between the Vineyard and New England signing communities, 1825–1893

After 1825, the integration of the Vineyard signing community into the wider New England signing community proceeded apace. Although the three eldest Mayhew siblings did not attend ASD, Lovey Mayhew and her younger brother Alfred did; most other deaf islanders did so too. Within 7 years, the first known marriage occurred between a deaf islander and a deaf mainlander: In April 1832, Mary Smith married Thomas Brown of Henniker, NH. Mary had finished her studies at ASD just 2-½ years earlier in November 1829. After their marriage, Mary lived in Henniker until her death in 1862 (Lane et al., 2000). The correspondence of Lydia Macomber (b.1811 in Westport, CT) shows that former ASD students visited Martha’s Vineyard as early as the 1830s.^11^ Lewis Perkins (who attended ASD 1832–1836) visited Deidamia Tilton in Chilmark in 1837; Mary Hillman (ASD 1829–1833) visited her in 1837 or 1838; and a teacher at ASD, Collins Stone, visited in the spring of 1838. William Turner visited sometime before 1847.

**Figure 13:**
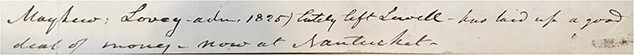
Excerpt from ASD’s “Alumni Book” recorded in June 1849 by Lewis Weld, ASD principal. *Note.* Used with the permission of the American School for the Deaf, Museum Archives.

In the 1840s, two more marriages were celebrated between deaf islanders and mainlanders: Benjamin Mayhew married Hannah Smith (ASD 1842–1843) in 1843 and Franklin Tilton married Sarah Foster (ASD 1838–1844) in 1847. Lovey Mayhew evidently spent time in the 1840s working in Lowell, MA and on Nantucket. Figure 13 shows an excerpt from a book kept by ASD’s administration that tracked former students (see Edwards, 2012). The excerpt reads: “Mayhew, Lovey. adm. 1825) lately left Lowell – has laid up a good deal of money – now at Nantucket.”

At least one hearing parent of deaf islanders visited Hartford: Captain Bartlett Mayhew had two deaf children, Mercy (1838–1859) and Jonathan (1843–1877). Mercy attended ASD at the same time as another deaf islander, Catharine Luce (1843–1857). Captain Mayhew brought both students to ASD in 1855. Catharine’s enrollment record states: “She came with Capt. Mayhew.” Later, Mercy wrote an essay, published in ASD’s 1855 annual report, expressing her desire, and perhaps that of Catherine and other fellow students, to remain at ASD for as long as possible: “We shall leave the Asylum and Hartford and we shall go home. We shall not return and visit Hartford perhaps and we shall be sorry that we shall not see it again. I shall be sorry to leave Hartford” (ASD, 1855, p. 29). As reported earlier, both young women died while at Hartford. Mercy is buried in Chilmark; in 1859, her father may have again journeyed to Hartford to bring her body home.

Among the early members of the New England Gallaudet Association were several deaf islanders. In addition to nine island residents who had attended ASD, Ruby Mayhew (1799–1876), who never attended a school for the deaf, was listed as a member of the Association in 1856 (Chamberlain, 1857). Thus, as the deaf island population was rapidly increasing in the 1840s and 50s, the Vineyard signing community was also integrating itself into the broader New England signing community (see Edwards, 2012, pp. 131–132).

This process happened quickly after the formation of the island’s signing community. By 1850, just 65 years after Benjamin Mayhew’s birth, most deaf islanders had attended ASD; hence, most would have spent years using emergent ASL. Several friendships and marriages led to sustained contact with mainland deaf individuals; and many deaf islanders were willing to pay the annual dues to maintain ties to the New England Gallaudet Association. Consider again the 11 deaf islanders born before 1825. The first offspring, whether deaf or hearing, of these individuals was a hearing boy (Benjamin Mayhew) born in 1839 to Sally (Smith) Mayhew. The first deaf child (Rebecca West) was born to Deidamia (Tilton) West in 1844. By 1839, six deaf islanders had attended ASD; in 1844, another three were in attendance. Benjamin, Rebecca, and other deaf-parented children who came after them were born into a signing community that had already been impacted by the New England signing community and by ASL.

If ASD had been established later, the Martha’s Vineyard signing community might have developed independently of any mainland signing community for a longer period. Or if similar demographic conditions had prevailed on, say, Ocracoke on North Carolina’s Outer Banks, a hypothetical deaf population there might not have been impacted so quickly by the early signing community in New England. Martha’s Vineyard was not remote enough in 1825 to remain isolated from the currents of deaf education and signing community formation in the U.S.

Compared to other well-studied cases, the integration of the Vineyard signing community into a larger signing community occurred relatively quickly. In Ban Khor, the first deaf individuals were born around 1933 and 1935, but it was not until roughly 50 or more years later, in the 1980s or 90s, that deaf Ban Khorians began to attend schools for the deaf and to have sustained contact with deaf individuals outside of their village (Nonaka, 2014). In Al-Sayyid, the first deaf individual was born in 1924, but access to deaf education for individuals in that community began some 60 years later in the 1980s (Kisch, 2012). In Adamorobe, Ghanaian Sign Language may have been introduced to deaf villagers in the 1950s—much more than a century after the first deaf individuals likely lived there (Kusters, 2012). In Bengkala, Bali, the first deaf individuals may have been born in the early 20th century (de Vos, 2016). De Vos (2012a) reported that several deaf individuals only recently had migrated away from the village—perhaps 100 years after the community’s founding.

In sum, signing communities vary in the span of time during which they develop independently of other signing communities. The Martha’s Vineyard signing community seems to have had a comparatively short period of independent development.

### Decline of the Martha’s Vineyard signing community

As the Vineyard signing community increasingly integrated itself into the New England signing community, the island-born deaf population was no longer reproductively isolated. This demographic change was crucial. The island-born deaf population mainly comprised homozygous carriers of Vineyard deafness. If deaf adults had married within the Vineyard deaf population, their children would likely have been deaf; this was true of the 1875 marriage of Freeman Smith and Deidamia West, which resulted in one deaf child and no hearing children. Even the children of deaf-hearing couples, in which the hearing partner was a heterozygous carrier, would have been deaf in two of four cases; witness the 1842 marriage of Deidamia Tilton (deaf) and George West (hearing), which resulted in five deaf children out of eight. However, after 1825, deaf individuals often married outside the Vineyard community; moreover, three young women of child-bearing age died while at ASD, and 14 deaf adults did not marry. Even at its peak in the 1850s, the deaf population on Martha’s Vineyard could not sustain itself.

Hearing islanders also contributed to, and experienced, demographic changes. Hearing–hearing island couples who were each heterozygous for Vineyard deafness were likely to have one deaf child in four births. Just as was true for the deaf population, island and mainland societies became more integrated in the 19th century due to improvements in travel to the island. Mainlanders immigrated to the island and some islanders moved away. The island’s population grew due to immigration while the local birth rate was likely decreasing, assuming Martha’s Vineyard followed broader 19th century trends. Here, we explore several factors affecting both deaf and hearing islanders that contributed to the decline in the island’s deaf population.

#### Births of deaf individuals to hearing parents

All 24 deaf children who were born between 1785 and 1843 were born to hearing parents. In 1844, Rebecca West was the first deaf child born to a deaf parent on the island since Beulah Lumbert’s birth in 1704. We saw in Figure 6 that the number of deaf births to hearing parents on the island declined in the second half of the 1800s: Just four deaf children were born to hearing parents in that period. Why did hearing islanders stop having deaf children?

In general, fertility rates in the U.S. and many other countries decreased across the 19th and 20th centuries, during what has been called the historical fertility transition (see Guinnane, 2011). Coale and Zelnik (1963) reported that the annual birth rate in the U.S. fell from 42.8 (per 1,000 white individuals) in 1855 (the first year reported) to 28.5 in 1900, a decline of 33%. In a study of fertility rates among Nantucket families from 1680 to 1840, Byers (1982) found that families in the 1780s had, on average, 4.8 children versus only 3.45 in the 1830s. With fewer pregnancies, there were fewer opportunities for heterozygous hearing parents to have deaf children.

Groce (1985) hypothesized that the opening of Vineyard society led to lower birth rates of deaf children. According to Banks (1911a, p. 30, citing federal census statistics), the island’s population increased by 40.6% from 1790 to 1900 (from 3,245 to 4,561); and from 1900 to 1950, the population grew by a further 22.2% (to 5,577; Martha’s Vineyard Commission, 2019, citing federal census statistics). The summer Methodist camp-meetings in the Wesleyan Grove at Oak Bluffs began in 1835 and, by 1858, drew more than 10,000 mainlanders to the island annually (Vincent, 1858). Immigrants from the mainland and Portuguese immigrants from the Azores, who came to the island at the height of the whaling era, are unlikely to have been carriers of Vineyard deafness. During this time, travel to and from the mainland became easier and more frequent with the advent of the first regular steam-powered ferry service in 1846 (Ewen, 2015). On the mainland, too, the increasingly developed rail network made it possible on August 7, 1875 for Alexander Graham Bell to leave Boston by train in the late morning for Woods Hole, take a steamer to the Vineyard, spend several hours on the island waiting for another steamer to Nantucket, and arrive there late that same day (Booth, 2021). Nonetheless, travel up-island to Chilmark remained difficult until roads were paved in the early 20th century (Dresser, 2019).

While the island was opening to mainlanders, some islanders also migrated to the mainland. For example, in the late 1840s, hundreds of island men took part in the California gold rush (Dunlop, 1996), and some would eventually settle in California. Bell’s list of deaf individuals who had “Vineyard ancestry” but who did not live on the island (see Figure S2 in the supplementary materials), includes two siblings, Frances and Mayhew Norton. Their hearing parents, who were both born on the island, separately migrated to California in the 1860s. Thus, these two deaf individuals were born to hearing parents who were apparently heterozygous for Vineyard deafness; the Norton siblings were separated from the Vineyard signing community by one generation. There may be other such births that Bell was unable to track at the end of the 19th century, and there may have been cases since. Unlike the Nortons’ parents, as islanders emigrated off-island, it became less likely that they would marry carriers of Vineyard deafness.

#### Decline in the deaf population

As we have suggested, one key factor leading to the decline in the Vineyard’s deaf population was the increase in marriages between island-born deaf and mainland deaf partners. In Table 3, we saw that 5 out of 21 of the offspring of these couples were deaf. We also saw that, although Hannah Smith was born in Maine, she is likely to have been a homozygous carrier of Vineyard deafness. Her 1843 marriage to Benjamin Mayhew on the island resulted in two deaf children and no hearing children. They married relatively late: Benjamin was 58 and Hannah was 34 in 1843. Had they married younger, more deaf children might have been born on the island.

In Table 4, we saw that many deaf islanders married hearing individuals. Although some of these hearing individuals are likely to have been heterozygous carriers of Vineyard deafness, just 7 out of 48 children born to these deaf–hearing couples were deaf. Sally Smith and Hariph Mayhew are both likely to have been carriers of Vineyard deafness. If we assume that Sally was homozygous and Hariph heterozygous, each of their offspring had a one in two chance of being deaf. However, the couple had just one child, who was not deaf. If they had had more children, perhaps more deaf children would have been born on the island.

The marriage rate of deaf islanders was lower than previously reported. We reported that 65% of island-born deaf adults married (26/40, see Table 2). That percentage is approximately 15% lower than the figure reported by Groce (1985, p. 78): “[A]bout 80 percent of those who lived to marriageable age did marry.” In part because of the relatively low marriage rate, the birth rate of the deaf population was also lower than previously reported. We calculated that the 28 deaf women born before 1900 had a birth rate (1.4; 0.4 deaf) well below the modern replacement fertility rate of 2.1. Of the 42 island-born deaf individuals born before 1900, two married and had no children, and nine married and had just one child. Groce (1985, pp. 79–80) reported that “Deaf Islanders had an average of 5.9 children.” We are unsure about the source of the discrepancy between these figures.

In sum, the decline in the deaf population on the island was furthered by the attendance at ASD of most deaf islanders, who found in Hartford a wider pool of potential partners than they would have had on the island. Many students from the Vineyard moved off-island for marriage or brought their spouses to the island after marriage. These relationships resulted in few deaf children. In addition, those deaf islanders who married hearing islanders had few children overall and few deaf children.

#### Decrease in the population of hearing signers

The relatively low marriage and birth rates of deaf islanders had direct consequences for the number of hearing individuals who could sign. As we have seen, 38 deaf-parented hearing individuals were resident on the island between 1692 and 2008. If the birth rate for the 29 deaf island women had equaled the contemporary replacement rate, there might have been 61 children parented by a deaf woman (of whom about 17 might have been deaf, using the actual ratio of deaf births to total births). Clearly, the deaf-parented population was much smaller than it might have been.

In Figure 3, we saw that the minimum age of the deaf population began to increase after 1860 and, with a few exceptions, continued to do so through 1950 and the years that followed. The increasing minimum age meant that there were fewer deaf children who might act as same-age signing peers for hearing islanders. Young hearing islanders from roughly 1860 on presumably had less access to sign than hearing islanders born earlier. Thus, the population of hearing signers likely aged along with the deaf population. We know from interviews with Gale Huntington that Catharine (Dolan) West, a deaf migrant from the mainland, provided childcare for the Huntingtons’ daughter (Huntington, 1983). We also know that Emily Poole taught her son several signs (e.g., “house,” “boat,” “cat,” and “dog”), which he used with a deaf adult (Poole, 1977). Hence some island children were exposed to sign despite the lack of near-age deaf peers. However, these intergenerational interactions would certainly have differed from the interactions that occur among peers.

These factors may have affected the signing community in another way. As more mainlanders immigrated to the island, the proportion of hearing signers in the community would have declined, even if the count of hearing signers did not change. Before moving to Chilmark, hearing migrants would have been unlikely to have had deaf and hearing neighbors who could sign. Not only would the sign language have been new to these newcomers, but likely also the culture of signing with one’s neighbors—what Groce (1985, p. 50) called “the island adaptation to deafness.” Gale Huntington was a hearing mainlander who joined the Chilmark community. In 1906, his family began summering on the island (Groce, 1985; Huntington, 1982). In 1915, when Gale was about 13, the family moved from New York City to settle permanently in Chilmark; at that point, there were about 10 deaf residents living in the town. Having settled in Chilmark in his teens, Huntington was not a fluent signer by his own report: “And of course, if they [i.e., the deaf residents] spelled it out I could get it, but if they were using the sign language, which was like a short hand, I could get some of it. But I couldn’t use it” (Huntington, 1982).

In this way, demographic changes to the Vineyard signing community may have shared similarities with more recent changes in Adamorobe and Ban Khor. Kusters (2012, p. 348) reported that the population of Adamorobe rapidly increased in the half-century between 1961 (pop. 405) and 2012 (pop. 3,500) and that villagers have “increasingly married immigrants.” According to Kusters, these demographic changes, in combination with a 1975 law that forbade deaf–deaf marriage, have resulted in fewer deaf births. Similarly, Nonaka (2014) reported that population growth since 2000 in Ban Khor has been driven to a large extent by immigration from outside the village. She concluded that “outsiders who marry into the village do not know [Ban Khor Sign Language] and are less likely to learn it than in the past…” (p. 62).

Different types of population growth may impact village signing communities in differing ways: Growth due to local births may increase the community’s core, while growth due to immigration may affect the demographic, genetic, and social conditions that initially supported the community’s formation. On Martha’s Vineyard, a decreasing number of deaf births coincided with an increasing number of immigrants to the island.

#### Impact of ASL on the Vineyard signing community

According to Nonaka (2014), contact with signers of Thai Sign Language, particularly through the presence of young Ban Khorians in residential schools for the deaf, has had the most important impact on the Ban Khor signing community—more important, even, than the demographic changes due to immigration described above. Ban Khor signers are shifting to Thai Sign Language, leaving Ban Khor Sign Language in an endangered state. What impact did emergent ASL have on the Vineyard signing community and on its language?

We have already seen that, beginning in 1825, contact with mainland signers at ASD profoundly affected the linguistic ecology of the Vineyard signing community. Nearly all deaf island residents attended ASD after 1825. Even the three eldest Mayhew siblings, who never attended any school for the deaf, had close connections to signers of ASL. After 1832, the Mayhews had an ASL-signing sister-in-law, who was also their second cousin: Sally Smith married their hearing brother Hariph. Benjamin Mayhew’s wife Hannah attended ASD for one year before their 1843 marriage. In 1850, the couple lived in a cluster of three houses in western Chilmark (see Figure 9), together with Elijah, Ruby, and two ASL signers—the Mayhews’ youngest siblings Lovey and Alfred.

By the late 19th century, the eldest Mayhew siblings had all passed away (Benjamin d.1852, Elijah d.1874, and Ruby d.1876); all other deaf individuals who were then living in Chilmark and West Tisbury had learned ASL at ASD, with three exceptions. As we noted, Josie West (1861–1945) never attended a school for the deaf. However, his mother and four older siblings were ASL signers; hence, the sign language that he acquired from birth was likely either ASL or a form of MVSL that had been strongly influenced by ASL. Likewise, Lavinia Smith (1877–1893) never attended a school for the deaf, but her deaf parents, both island-born, had attended ASD and were ASL signers. George Trask (1880–1891) never attended a school for the deaf, but both of his parents had attended ASD. Thus, all deaf members of the Vineyard signing community either learned ASL at ASD or had close connections to multiple individuals who had done so. It seems therefore likely that many deaf island residents began to shift from MVSL to ASL during the 19th century.

However, village signers do not always completely shift from their village sign languages when exposed to a national sign language. Ergin et al. (2018, p. 636) reported that there were four signers (two pairs of siblings) of Central Taurus Sign Language (CTSL) who were exposed to Turkish Sign Language (TİD) while attending schools for the deaf outside their village. These signers reportedly “use a mixture of CTSL and TID among one another but they switch to CTSL to communicate with everyone else in the village.” One important difference between this signing community and that of the Vineyard is that all deaf individuals living in the Turkish village were born there. In contrast, the Vineyard signing community hosted mainland deaf individuals starting in the 1840s and hence may have felt greater pressure to use ASL.

What about the hearing members of the Vineyard signing community? We know that hearing signers in village signing communities may sometimes maintain a community’s sign language even after deaf signers have shifted to another sign language. Lanesman and Meir (2012) reported that many deaf individuals, and entire deaf families, completely shifted from Algerian Jewish Sign Language to Israeli Sign Language after immigrating to Israel. These deaf individuals attended Israeli schools for the deaf and became integrated into the signing community in that country. In contrast, hearing signers in the Algerian Jewish signing community had few connections to Israeli signers and continued to use Algerian Jewish Sign Language with their deaf family members.

Although limited linguistic data have been reported about MVSL (Bahan & Nash, 1995; Nash, 2015; Orfila, 2024), some evidence suggests that hearing members of the Vineyard signing community acquired features of ASL. We have already seen that Gale Huntington reported knowing how to use ASL’s fingerspelling system; interviews with Huntington recorded in 1977 confirm that point. Similarly, video-recorded interviews show that other hearing islanders, such as Eric Cottle, were fluent fingerspellers—even decades after the last deaf residents of Chilmark had died in the 1950s. Bahan and Nash (1995, p. 15) concluded: “[MVSL], as it was used in the late 1800s, was very similar to the sign language used on the mainland. By that time many of the deaf people had attended ASD and brought back signs and other elements of what was now American Sign Language, which were passed along to other community members.”

Nash’s and Groce’s early notes from interviews with hearing islanders indicate that many of their signs were similar to ASL (see Orfila, 2024). While some hearing islanders have said in interviews that they were unable to understand mainland signers, others apparently were able to do so. Consider this excerpt from a 1977 interview with Emily Howland Poole, a hearing islander (see Bahan & Nash, 1995, pp. 15–16).

EHP: “Your great grandfather [Everett Poole, a hearing islander] and I were on... a sightseeing trip in Tennessee. And sitting behind... us were two ladies and they were deaf and dumb... So he turned around and said ‘How do you do?’ Well they started up a conversation and they talked all that trip....”

Interviewer: “Was their sign different than what you learned?”

EHP: “No, no. I don’t think so. Because my husband of course... talked the Martha’s Vineyard deaf and dumb language, you might say. And maybe, maybe they had different signs. I don’t know. But I know they carried on that conversation.” (Poole, 1977).

How did hearing islanders come to acquire features of ASL? In rural Chilmark and western Tisbury, hearing signers interacted with island-born deaf signers and, beginning in the 1840s, with mainland deaf signers who had acquired ASL at ASD but who had not grown up with MVSL. In this way, the Vineyard signing community may have differed from the Algerian Jewish signing community in Israel. In that urban community, social networks were more distinctly demarcated: Hearing signers interacted with their deaf Algerian Jewish family members and neighbors, but rarely did so with signers of Israeli Sign Language (Lanesman & Meir, 2012). In the rural Vineyard signing community, the social networks of hearing and deaf community members, both islanders and mainlanders, were highly interconnected.

Another difference between the Vineyard signing community and some other village signing communities may have facilitated communication between deaf and hearing islanders—namely, high rates of literacy and thus bilingualism. Literacy in English was an important pedagogical goal at ASD (Edwards, 2012). By the time deaf islanders had finished their studies in Hartford, they were likely able to read and write in English (Groce, 1985). Because nearly all deaf islanders attended ASD, the proportion of literate deaf Vineyarders may have been higher than the proportion of literate deaf residents of many other village signing communities. For example, from 1933 to the 1990s, deaf Ban Khorians did not attend deaf schools, and most had limited literacy in Thai (Nonaka, 2014). Similarly, in Bali, deaf individuals have only recently begun attending deaf educational programs (de Vos, 2012b).

Since most deaf residents of Chilmark were bilingual, those hearing islanders who learned to fingerspell, like Huntington and Cottle, could communicate with their deaf neighbors, even if the hearing individuals had limited knowledge of ASL or MVSL. Then, as now, the ASL fingerspelling system had just 26 handshapes, and it was based on English, a language hearing islanders already knew. In another excerpt from the interview with Poole, she indicated that she never had extended conversations with deaf islanders; nevertheless, even in her 90s, she was able to remember most of the fingerspelling alphabet.

EHP: “So I walked out and stopped him [Jared Mayhew, a deaf islander]. And he stopped – and we said, ‘How do you do?’ Then I said, ‘My husband told me to tell you over boat house codfish for you’. And he said, ‘I know’. I think that was the longest conversation I ever had with a deaf and dumb person. Oh, they were grand people.”

Interviewer: “Do you remember the alphabet?”

EHP: “All but... let’s see... A, B, C, D, E – I’m not sure of – F, G, H, I, J, K, L, M, N, O, P, Q, R, S, T, U, V, W, X, Y... is that right?” (Poole, 1977).

Other interviews with hearing islanders indicate that fingerspelling was a frequent mode of communication between the deaf and hearing. Consider this excerpt from a 1983 interview with a hearing resident of Chilmark named Gladys (Mayhew) Flanders.

GF: “I used to talk, I can’t now because they have new signs and new ways but we used to just spell and make ordinary signs. See, A, B, C, D, E, F, like that you know and talk to them.”

Interviewer: “Were there some words, some particular Vineyard words and sayings?”

GF: “No, I don’t think so. You most always spelled what we wanted to say to them.”

Interviewer: “So, you could converse with them just like a person that could hear then?”

GF: “Yes, they would spell back to you.” (Flanders, 1983)

In its fingerspelling system, at least, ASL seemingly had an important impact on the Vineyard signing community. Because not all hearing islanders were fluent signers, the fingerspelling system opened a communication channel between them and their deaf neighbors.

## Conclusion

The Martha’s Vineyard signing community arose in a relatively remote part of Chilmark, the least populous town on the island. From 1785 to 1817, the community’s deaf population was concentrated in just two families. In 1825, only 8 years after ASD had opened in Hartford, the first deaf islanders enrolled there. From that year on, the fledgling Vineyard signing community began to integrate itself into the nascent New England signing community. Conversely, the island community began to integrate deaf mainlanders, most of whom had married into the community. Through this process of bi-directional integration, the conditions that initially supported the formation of the island’s signing community began to change. The deaf population, comprised in the main of homozygous carriers of Vineyard deafness, was no longer reproductively isolated. Broader social changes and transportation innovations meant that heterozygous hearing individuals were, similarly, less isolated than theretofore. The sign language that had begun to develop before 1825 came into intense contact with emergent ASL and with English. By 1850, close to the peak of the island’s deaf population, most deaf islanders had attended ASD and learned ASL. Many may have begun to shift to ASL. High rates of English literacy among deaf islanders may have facilitated communication with their hearing neighbors, many of whom learned to fingerspell from them. As deaf islanders began to shift to ASL, the close social networks that developed in this rural community may have facilitated hearing members’ adoption of features of that sign language.

As one of the first village signing communities to have been studied, the Martha’s Vineyard community has rightly fascinated scholars and public audiences (e.g., LeZotte, 2020) in the decades since Groce’s 1985 publication of *Everyone Here Spoke Sign Language*. Much earlier, the island’s deaf population had attracted the attention of ASD faculty members, Alexander Graham Bell, and the artist Thomas Hart Benton, and had inspired sensationalist accounts of a “peculiarly afflicted people” (“Mark of Chilmark,” *Boston Sunday Herald*, January 20, 1895). Despite the attention paid to the island’s deaf population since the 1840s, it is only since the 1970s, with the research of Joan Nash and Nora Ellen Groce, that the Vineyard’s signing community and sign language have come into focus. Earlier sources are silent about the language that deaf islanders used and about whether that language differed from ASL on the mainland. If, as has been suggested (Groce, 1985; Lane et al., 2000; Nash, 2015), deaf islanders came to ASD using a fully formed sign language that differed considerably from emergent ASL, it seems strange that there is no mention of that fact in ASD’s enrollment records, in its annual reports, in the numerous essays written by ASD students in those reports, in 19th century accounts of deaf islanders published in the *American Annals of the Deaf* (Bell, 1886), or in other reports by Alexander Graham Bell, who was a signer and who had spent time working at ASD as an instructor of teachers of articulation (Bruce, 1973).

Part of the reason for this silence in earlier sources is likely due to 19th century notions of the *natural* sign language (Baynton, 1996). Because signing everywhere was thought to be universal, differences in the sign languages used in, for example, France, Germany, and the U.S. were likened to regional dialectal differences of the same language (Peet, 1853). From that perspective, it is unsurprising that a focus on the distinctive history of the Vineyard signing community appeared only in the late 20th century, after scholars had begun to understand that signing communities and their languages have their own particular histories (see Power, 2022).

Our study yields an additional reason for the silence in the 19th century historical record. If, as we have argued, the island’s signing community began to form only in 1785, then the community had had only a short period of independent development before it came into contact with the nascent New England signing community. When the first islanders arrived at ASD in 1825, their language—having primarily emerged by then in just two or three families—may not have seemed any more distinctive than the homesign systems that had emerged in other parts of New England. Thomas Brown from Henniker, NH, whose father and sister were deaf, was a second-generation homesigner when he enrolled at ASD in 1822 at age 18 (Lane et al., 2000). Compared to the Browns’ homesign system, which had begun to emerge with the birth of Thomas’ father in 1772, MVSL had had a shorter period of development. In just the 1825 school year, the two Derby sisters of Weymouth, MA enrolled at ages 14 and 15; two Smith siblings (ages 15 and 16) enrolled from Hawley, MA; and on October 26, 1825, the day after the first three Vineyard students arrived in Hartford, four Packer siblings (ages 16, 21, 22, and 24) arrived from Leyden, MA. Compared to the Packers’ homesign system, which had begun to develop in one family in 1801, MVSL had had just 16 more years of development.

As we have shown, just as the island’s deaf population and its signing community began to flourish, members of that community also began to integrate themselves into the New England signing community through education, marriage, visits to the mainland, and membership in the New England Gallaudet Association. Our analysis of marriage patterns reveals a community that had become well connected to deaf mainlanders by the peak of the island’s deaf population in 1857. As increasing numbers of deaf islanders attended ASD and as deaf mainlanders married into the island community, the distinction between island and mainland signing communities weakened. Even hearing signers on the Vineyard were influenced by these changes; many learned features of ASL, such as its fingerspelling alphabet and many of its signs.

Our reinterpretation of the growth and decline of the Martha’s Vineyard signing community allows us to now reconsider Lane et al.’s (2000) distinction between assimilating and differentiating societies. On their account, differing genetic patterns of deafness may lead to differing types of societies. As in Chilmark, a widespread recessive gene for deafness within a reproductively isolated population may lead to numerous deaf individuals who are interspersed throughout that population. Deaf and hearing individuals share close-knit social networks in such assimilating societies, and many hearing individuals in those societies may learn to sign. Differentiating societies, in contrast, may form due to dominant patterns of deafness. As in Henniker, NH, if deafness is restricted to one family, hearing members of society may have few deaf contacts within their social networks and hence few opportunities to learn to sign. According to Lane et al., in a differentiating society, deafness is passed down the generations as a heritage, and a distinctive deaf identity may develop.

Our work on the Vineyard signing community blurs this contrast. Following Groce, Vineyard deafness was likely due to a widespread recessive gene. Yet, even before 1825, Lovey Mayhew “often asked” her father to allow her to attend ASD. Evidently, even in Chilmark, the seeds of a Deaf identity had begun to form. In 1832, just 2-½ years after finishing her studies at ASD, Mary Smith married a deaf mainlander and moved to New Hampshire. That same year, Mary’s sister, Sally, married a hearing islander. Thus, in the same family we find evidence for both types of societies. In later decades, deaf islanders would marry deaf and hearing individuals from both the mainland and the island and would keep in touch with their mainland deaf friends. Deidamia Tilton, who married a hearing islander, was visited on the island by her former schoolmates. Those who had attended ASD would visit Hartford for events attended by hundreds of deaf New Englanders. Even islanders like Ruby Mayhew who did not attend ASD would become members of the New England Gallaudet Association.

The Vineyard signing community therefore reflected aspects of both assimilating and differentiating societies. Groce’s work (as well as that of Joan Nash and Linsey Lee) has shed light on the integration of deaf islanders into island society, particularly in Chilmark and western Tisbury. Their scholarship (and that of Lane and colleagues) has emphasized the distinctive aspects of the island community, such as MVSL vocabulary and signing practices that were distinct to the island. Our reinterpretation of the Vineyard signing community situates it within the wider history of 19th century New England, including the founding of ASD and the formation of the New England signing community. Deaf islanders both integrated themselves into this nascent community and welcomed deaf mainlanders to Martha’s Vineyard.

## Supplementary Material

Supplementary_material_enad058

## Endnotes

1
Nash (2015) reported that a deaf child was born into the Wampanoag tribe on the island in the 1950s but was not involved in the Vineyard signing community. For that reason, and for reasons of privacy, we have not included this individual in our analyses.

2 We do not know exactly when George Trask (1880–1891) moved to the island. We assume in this analysis that he moved in June 1880. However, if he moved after March 18, 1881 (i.e., the date that was 10 years before his death), then he also would have lived for fewer than 10 years on the island.

3 We have not attempted to identify the children of the three deaf islanders born after 1900 because of privacy concerns and because U.S. census records are only available through 1950.

4 According to ASD’s records, Sabrina was “born deaf,” and “her mother [was] frightened by an idiot before her birth.” Although Lane et al. (2011, p. 100) report that “she was from a large Deaf clan,” Sabrina was apparently not related to the Rogers family whose lineage is shown in their book on page 225.

5 Wadsworth and Jerusha Mayhew were distantly related to Benjamin Mayhew; all three were descendants of Thomas Mayhew (b.1620–1621), who moved to New England in 1631 (Banks, 1925).

6 Rebecca Skiff may have been the unidentified deaf woman in Lydia Mayhew’s report in Turner (1847). The timing of the funeral, likely in 1784, fits with Banks’ (1925, p. 315) report that Rebecca’s children, born in 1781 and 1784, died young. Although Banks does not indicate where the children were born or died, Rebecca's 1779 marriage to Josiah Mayhew took place in Chilmark. Perhaps the couple still resided there in 1784. However, it seems unlikely that Rebecca was then a Chilmark resident because Lydia, who was born in Chilmark, had never previously met her. Similarly, the other mothers, Sarah (Cottle) Smith and Rebecca (Tilton) Tilton, who were also born in Chilmark, had never met her. We note that one member of Rebecca’s family, her husband’s sister Reliance, moved to Maine in 1791; she died in New Sharon, ME, in 1808.

7 We are unsure about the number of deaf-parented hearing individuals born off-island in the 20th century.

8 We have found no marriage records for 23 of the 89 hearing individuals who had deaf siblings and who did not have a deaf parent. An additional eight did not marry.

9
Fay (1898, p. 6) cited statistics in Bell (1884), who found a marriage rate of 45.5% for former students of ASD and of the Illinois School for the Deaf who were born before 1840 and who had married up to 1882. It would be incorrect to assume that the marriage rate of that sample was representative of all 19th century deaf mainlanders.

10
Fay (1898, p. 24) noted that deaf–hearing marriages were likely undercounted because of the survey’s sampling method, which in part relied on schools for the deaf to locate former students who could provide information about their marriages.

11 Lydia Macomber's ``letter book'' can be found at the Westport Historical Society's website: https://wpthistory.org/2014/11/lydia-macomber/.
