# Supplementary material for: The historical demography of the Martha’s Vineyard signing community
Source: J Deaf Stud Deaf Educ. 2024 Jan 29;29(3):295–321. doi: 10.1093/deafed/enad058 (PMC11195467; doi:10.1093/deafed/enad058)
Supplement: Supplementary_material_enad058 [file supplementary_material_enad058.pdf]

Sources and Methods

Here we outline the sources that we used to understand the deaf population and the signing community on Martha's Vineyard. We also describe the databases of deaf and hearing islanders that we have compiled. Finally, we describe challenges that confront research on the deaf population in the U.S. before 1850, and how we addressed those challenges.

Sources

There are abundant historical and genealogical records pertaining to the residents of Martha's Vineyard during the period relevant to this study. Although many of the sources mentioned below are available online, others can only be accessed in libraries or archives. We visited the archives of the American School for the Deaf in Hartford, CT, the Martha's Vineyard Museum, the Gallaudet University Archives in Washington, DC, and the archives of the Alexander Graham Bell Association in Chantilly, VA. We also consulted the Alexander Graham Bell Family Papers in the Library of Congress;[1] only selected items from the Bell Family Papers have been digitized.

Eighteenth-Century Records

The earliest reference to a deaf resident of Martha's Vineyard is found in the *Diary of Samuel Sewall* (Sewall, 1879, p. 432) in which Judge Sewall recounts his meeting with "Jonathan Lumbard" on 5 April 1714 near Oukakemy Bay (now, Lambert's Cove).[2] This Jonathan Lumbard may have been the same individual who was identified in contemporaneous Tisbury Town Records as "Jonathan Lumberd" and, most frequently, "Jonathan Lumbert." Although Jonathan Lumbert's name is found repeatedly in Tisbury's early town records (Swift & Cleveland, 1903),[3] Sewall's diary yields the only extant indication that Lumbert was deaf—or, in Sewall's (p. 432) words, "deaf and dumb." Prior scholars have understood the reference in

Sewall's diary to identify the Jonathan Lumbert (often "Jonathan Lambert" in the secondary literature: e.g., Groce, 1985; Lane, Pillard, & Hedberg, 2011) who was born in 1657 in Barnstable, MA and who moved to Martha's Vineyard in the early 1690s. However, when Sewall traveled to the island in 1714, there were two Jonathan Lumberts resident in Tisbury—the elder Jonathan, who was then 56, and his son, who was 29. In our view, we cannot be certain whether Sewall meant the elder or the younger Jonathan because neither are identified as deaf in any other primary sources.

Two additional individuals, both members of Jonathan Lumbert's family, may have been deaf. In Jonathan's last will and testament, probated in 1738 in Tisbury, he refers to "my two poor children that cannot speake for themselves."[4] Although Lumbert does not identify these two children by name, it has been thought that this part of the will refers to Ebenezer and Beulah Lumbert, who were both apparently unmarried adults when Jonathan Lumbert's will was written; the phrase "cannot speake for themselves" has been thought to identify these two individuals as deaf (Banks, 1911b; Groce, 1985). However, Ebenezer and Beulah are not explicitly identified as being "deaf" or "deaf and dumb" in any extant historical record. Moreover, the phrase "cannot speake for themselves" is not used to identify any other individual on the island as deaf; hence it seems possible that Ebenezer and Beulah were not deaf. The quoted phrase may have been a legal reference to these individuals' competence to represent themselves.[5] The question of Ebenezer and Beulah's deafness remains unresolved, in our view.

Apart from the records pertaining to the Lumbert family, just one other individual is identified as "deaf" (i.e., hard-of-hearing in contemporary parlance) in a primary source of the 18th century: Wadsworth Mayhew, a resident of Chilmark until 1777 (Banks, 1925), is so identified in Volume 1, Tort 1 of the *Legal Papers of John Adams* (Wroth & Zobel, 1965).[6] The

incidents involving Wadsworth Mayhew began in June 1762; Adams traveled to Martha's Vineyard in August 1765 to participate in the trial as an attorney (Adams, 1765).

Nineteenth-Century Records

Beginning in the 19th century, primary sources increasingly refer to the hearing status of individuals on Martha's Vineyard. Some of these records were produced on the island. Between 1761 and 1827, two clergymen (Samuel Kingsbury and Joseph Thaxter) recorded the deaths of residents of Edgartown; among those deceased residents was George Corliss Pease (spelled "George Corlis Peaſe"), who drowned in the English Channel in 1801, and was identified as "deaf and dumb" (Pease, 1905).

On 12 June 1817, the year ASD was founded in Hartford, the General Court of Massachusetts passed a resolution entitled, "Resolve to ascertain the number of deaf and dumb persons, throughout the state" (General Court of the Commonwealth of Massachusetts, 1819). In response, two selectmen of Chilmark (Matthew Mayhew and John Hancock) reported the age and gender of seven deaf residents of that town from two families: Three males and two females (ages between 14 and 32) in one family and two females (ages 7 and 12) in another. A decade later, a Massachusetts House Order of 11 June 1827 required a second census of the state's deaf residents. On 21 January 1828, the selectmen of Chilmark reported that they had counted 11 deaf residents (6 males, ages 3 to 42; and 5 females, ages 9 to 28). In a letter dated 18 September 1827, the selectmen of Tisbury reported that "there is not one [deaf and dumb person] within the limits of the town of Tisbury."[7] Only Chilmark responded to both the 1817 and 1827 resolutions. Tisbury submitted a return—the letter just mentioned—in 1827. Edgartown did not submit returns for either 1817 or 1827.

In 1860, an anonymous resident of Chilmark wrote a letter to the editor of the *Vineyard Gazette*; the writer reported that "within 50 years, there have been eight families in which deaf and dumb children have been born" (Anonymous, 1860).

In addition to records produced on the island, deaf islanders were mentioned in records produced by ASD in Hartford and by early deaf associations. ASD recorded the enrollments of 25 students from Martha's Vineyard—all between 1825 and 1896; these enrollment records typically noted the names, birth order, and hearing status of students' siblings, as well as the hearing status of parents and other relatives, such as aunts and uncles (Power & Meier, 2023). The school's annual reports, which are in part derived from the enrollment records just mentioned, are available online via Google Books. The school kept records of the whereabouts of alumni in its *Record of Facts Concerning the Former Pupils of the American Asylum*. It also kept records of attendance at large events in a *Record of the Names of Those Present at Several Gatherings of Deaf Mutes at the American Asylum*; those gatherings took place in 1850, 1854, 1860, and 1866. Several deaf individuals from Martha's Vineyard were recorded both in the school's *Record of Facts* and among the attendees in the *Record of the Names*. Deaf island residents appeared in the membership lists of the New England Gallaudet Association, an early deaf-led organization and forerunner to the National Association of the Deaf (e.g., New England Gallaudet Association, 1854).

William Turner, an instructor at ASD, travelled to Martha's Vineyard at some point in the early 1840s.[8] In Chilmark, he interviewed the parents of several former students (Turner, 1847). In addition to these first-person accounts, the correspondence of Lydia Macomber, who was a student at ASD from 1832 to 1836, describes how two of Lydia's former schoolmates traveled to Chilmark in 1838 to visit Deidamia Tilton, another former schoolmate and resident of the

island.[9] Finally, a letter addressed to Alexander Graham Bell from the Massachusetts Office of the Inspector of Charities (dated 8 August 1884) identified all families with deaf individuals on the island known to that office; that letter is available in the Alexander Graham Bell Family Papers at the Library of Congress.

Census and Genealogical Records

Beginning in 1830, the decennial U.S. federal census recorded the hearing status of residents; the Massachusetts state censuses of 1855 and 1865 did the same for residents of that state. The 1830 and 1840 U.S. censuses reported the names of heads of households, as well as the sex, race, and age bracket (under age 14, between 14 and 25, and above 25) of each individual living in a household. Individuals were also reported as “deaf and dumb”, blind, or foreign. Thus, in those two federal censuses, a deaf individual who was not the head of any household was not specifically identified by name, although many other characteristics of those individuals, such as age and sex, were reported. From 1850 until 1880, the federal census records identified all individuals by name; the 1855 and 1865 Massachusetts state censuses did the same. In 1900 (and subsequent censuses), the Census Bureau did not inquire about deafness.[10] *The Special Census on Deaf Family Marriages and Hearing Relatives, 1888–1895*, which was published in 1898 as a supplement to the 1890 federal census, included information about the marriages of deaf residents of Martha’s Vineyard and their offspring (Fay, 1898).

In addition to these census records, numerous other genealogical records—such as the birth, marriage, and death records of island residents—are available in online repositories (e.g., Ancestry: ancestry.com; and Find A Grave: findagrave.com). Much of our understanding of the genealogies of island residents and the locations of their homes is due to Richard Pease (1814–1888), who unofficially served as the island’s genealogist. Pease was also Assistant Marshal for

the 1850 federal census. It was Pease who found, and copied down, the records of Edgartown deaths in the notes of Kingsbury and Thaxter mentioned above (H. Pease, 1905). Around 1858, he recorded the locations of households in Chilmark on Walling's (1858) map of Martha's Vineyard. Pease's research forms the basis of the extensive genealogical information in Banks (1925) and in the work of Alexander Graham Bell (see Banks, 1911a, pp. 7–8). In 1885, Pease agreed to provide his genealogical research to Bell,[11] who was interested in the island's deaf population as part of his broader research on the inheritance of deafness and on pedagogical methods in deaf education (Booth, 2021; Gordon, 1892).

Bell produced voluminous research on the genealogies of Vineyard families, particularly those families with deaf individuals in their lineages. To date, the greater part of his research has remained largely unknown (cf. Groce, 1985; Lane et al., 2011) and relatively inaccessible in the Alexander Graham Bell Association's archives. Bell visited the island in 1885 and 1887 (Booth, 2021), and apparently interviewed members of at least one household in Chilmark, the West family, which included six deaf individuals (Anonymous, 1931).[12] In the 1880s, his research on the island's deaf population attracted the attention of a wide audience. In November 1883, Bell (1884) presented his research on deaf heredity to the National Academy of Sciences.[13] In June 1888, he was invited to testify before a Royal Commission of the United Kingdom, as was Edward M. Gallaudet (Gordon, 1892). Deaf individuals from Martha's Vineyard were featured in both presentations. In 1887, Bell produced what he considered to be an exhaustive list of all 72 deaf individuals "known to be descended from Martha's Vineyard people"; see the section Alexander Graham Bell's List of 72 Deaf Individuals. Bell's previously unpublished list includes deaf individuals who were either born on the island (44) or who lived off-island and had island

ancestry (28); the list additionally includes six deaf individuals from off-island who had married one of the 44 deaf island residents.

Deaf Individuals Identified in Secondary Sources

Until the mid-19th century, there were few systematic attempts to identify deaf individuals in the U.S. We have already described the deaf censuses ordered to be taken in Massachusetts in 1817 and 1827, the federal censuses of 1830 and 1840, and ASD's enrollment records. In addition to these primary sources, there is now a rich secondary literature about the deaf population of Martha's Vineyard—most importantly, work by Bell, Groce (1985), and Lane et al. (2011). In a relatively small number of cases, individuals have been identified as deaf in the secondary literature, but we have been unable to find any corroborating primary sources; see Table S1. Here we describe those individuals whose deafness remains uncertain. See above and the section Jonathan Lumbert for our discussion of uncertainties surrounding deafness in the Lumbert family.

<Insert Table S1 here.>

There are several individuals who are identified as deaf in only one source. For example, in genealogical charts accompanying his list of "72 Deaf-Mutes of Martha's Vineyard," Bell identified Jerusha Mayhew as "deaf," Rebecca Skiff and Hannah Skiff as "deaf and dumb," and Andrew Butler as "mute." We have been unable to find any dispositive evidence about these individuals. Nevertheless, we include all but one of them in our analyses here, based on the assumption that Bell's research was itself based on Pease's genealogical research in the 19th century. Pease likely had access to oral history and, perhaps, documents that are no longer available to us. We cannot include Hannah Skiff in our analyses because we have found no records about her.[14]

In our analyses, we have not included the three individuals identified in Table S1 who are found only in Lane et al. (2011) but not in other secondary sources. Lane et al. identified Benjamin Lambert as deaf; Benjamin was the son of the younger Jonathan Lumbert. They also identified Jerusha Tilton as deaf (see also Lane et al., 2000, p. 23) as well as one Zeno Tilton, who died in 1817.[15] These individuals were not identified as deaf by Bell, and we have not been able to find any evidence indicating that they were deaf, despite having carefully reviewed Lane's papers at the Gallaudet University Archives.[16] Finally, Groce (1985, p. 42) reported that there were six deaf individuals alive in Edgartown in 1800, but we have only identified two. In reference to a shipping accident that cost the lives of two brothers, one deaf and one hearing, Groce (p. 83) identified a family of four deaf individuals alive on the island in the early 19th century. It is possible that these four individuals were four of the six deaf individuals mentioned by Groce as living in 1800, but we have not yet been able to confirm that hypothesis. As we have seen, Edgartown did not provide a census return in 1817, likely indicating that there were no deaf individuals then residing in that town. If Groce was correct about the four deaf individuals in Edgartown in the early 19th century, they had evidently left by 1817.

Methods

Identifying Deaf Individuals in Census Records Prior to 1850

As discussed above, only the head of a household was identified by name in federal censuses prior to 1850. In the 1830 and 1840 censuses, the number of deaf individuals in each household was recorded in a table that was separated from (across a left-right page break), but linked to, the names of heads of households. Although this reporting method complicates the task of identifying deaf individuals by name, in practice it was relatively straightforward to identify nearly all the individuals identified as deaf in the 1830 and 1840 censuses on Martha's Vineyard.

Starting from the 1850 federal census, in which deaf individuals were identified by name, and working backwards in time, we identified deaf individuals across earlier records using other information reported about each individual, such as age, sex, and household of residence. Here we explain how we did this for the towns of Tisbury and Chilmark.

Tisbury. In the 1850 federal census, the inhabitants of Dukes County were reported in three towns: Chilmark, Edgartown, and Tisbury. In this section, we focus on the five deaf residents reported in Tisbury in 1850. The census reported, inter alia, each inhabitant's name, age, sex, occupation, and place of birth, as well as whether an inhabitant was deaf. Any deaf individual above the age of 10 in 1850—there were three such individuals in Tisbury—should have been reported in the 1840 census; by the same reasoning, any deaf individual above age 20 in 1850—one in Tisbury—should have been reported in the 1830 census.

The three deaf residents of Tisbury above the age of 10 in 1850 were Mercy Mayhew (11) in the household of Bartlett Mayhew, Almira Luce (18) in the household of Ulysses Luce, and Sally (Smith) Mayhew (43) in the household of her husband, Hariff (or Hariph) Mayhew. In 1840, the entries for each of these three households list one deaf female in the expected age range: one female under age 14 in the household of Bartlett Mayhew, one female under 14 in the household of Ulysses Luce, and one female over 25 in the household of Hariff Mayhew. Two others are identified in the 1840 census in Tisbury as being "deaf and dumb": one male under 14 in the household of Moses Cromwell[17] and one female under 14 in the household of Jabez Luce. The son of Moses Cromwell, Isaac Cromwell (b.1831), was apparently not deaf; instead, he was reported in the 1850 federal census to have had some type of intellectual disability, using the now-anachronistic term "idiot." It remains unclear who the deaf female was in Jabez Luce's household in 1840; no deaf individuals were reported in that household in either 1830 or 1850.[18]

No deaf individuals were reported in Tisbury in 1830. This is expected; of the deaf individuals living in that town in 1850, only Sally Mayhew was alive in 1830. However, she did not marry until 1832 and resided in Chilmark with her parents in 1830. As discussed above, prior to 1830, there were no deaf individuals reported in Tisbury in the censuses of 1817 and 1827.

Chilmark. Here we show how we connected the deaf residents of Chilmark who appear in the 1850 census to the deaf individuals identified in censuses before 1850. Beginning in 1850, the federal census listed all individuals by name. In Chilmark that year, there were 17 deaf residents. See Table S2.

<Insert Table S2 here.>

All individuals in Table S2 over age 10 should have appeared in the 1840 federal census and, conversely, the four individuals under age 10 should not have appeared in that census. In addition, we would not expect to find Hannah Mayhew or Sarah W. Tilton in the 1840 census because both women moved to the island in the 1840s. Based on this reasoning, we would expect to find 11 deaf residents of Chilmark in 1840. In the census of that year, there were indeed 11 deaf residents in Chilmark; see Table S3. The census reported, inter alia, the name of the heads of household and the age bracket of deaf individuals living in each household.

<Insert Table S3 here.>

In 1830, by the same reasoning, we would expect to find all individuals who were over the age of 10 in 1840. Only Prudence Lambert was under age 10; hence we would expect to find 10 deaf residents of Chilmark in the 1830 census. In fact, there were 12 deaf residents in 1830; see Table S4. The two additional deaf individuals were Sally and Mary Smith. These two women moved away from Chilmark in the 1830s; hence they were not in Chilmark for the 1840 census.

Sally Smith married Hariph Mayhew and moved to West Tisbury; and Mary Smith married Thomas Brown and moved to Henniker, NH.

<Insert Table S4 here.>

Based on their ages in 1830, we would expect to find all but one of the individuals in Table S4 in the selectmen report dated 21 January 1828. As expected, the selectmen noted that there were 11 deaf residents of Chilmark living in four households; see Table S5. The report did not name the heads of household or the deaf individuals; but it did list each deaf resident's age and gender.

<Insert Table S5 here.>

Finally, the 1817 selectmen report (dated 31 December 1817) listed seven deaf residents of Chilmark in two families, as expected; see Table S6. The report noted only the genders of deaf individuals in each family and the ages of the youngest and oldest deaf individuals.

<Insert Table S6 here.>

Analyses

Using the sources described above, we created several related data sets with demographic information about deaf island residents and their families. We describe these linked data sets below. We analyzed the information in our database using Python (Van Rossum & Drake, 2009) and the Pandas library for data analysis (McKinney, 2010). We used SciPy (Virtanen et al., 2020) and NumPy (Harris et al., 2020) for statistical analyses. Figures were produced using Matplotlib (Hunter, 2007), Plotly (Plotly, 2015), and Geopandas (Jordahl et al., 2020).

We created one database of all 53 deaf individuals who were resident on the island between roughly 1692 and 1998. We compiled genealogical records of these 53 individuals' births, deaths, and marriages, as well as records identifying them as deaf. We recorded the dates

during which deaf islanders were resident on Martha's Vineyard, including the sources for these dates; we also recorded the towns in which deaf individuals were resident. Lastly, we recorded information about the marriages and children of deaf islanders.

Based on our database of deaf island residents, we searched genealogical records, the genealogies in Banks (1925) and Fay (1898), and ASD's enrollment records for information about the hearing family members of deaf islanders. We created a second database with information about these family members; this information included, when available, their births, deaths, and marriages. We identified 201 hearing family members of the 53 deaf individuals mentioned above, including their parents, siblings, children, and spouses. Of these 201 hearing family members, 182 resided on the island; the other 19 lived off-island. For some of these hearing family members, we were unable to locate dates of their births (two island residents), deaths (47 individuals, 43 island residents), or marriages (52 individuals, 42 island residents). Of the 52 individuals for whom we have no marriage dates, 21 (16 island residents) did not marry; for another six (five island residents), we were only able to identify their spouses. These 52 individuals, plus one for whom we have no birthdate, were not included in the marriage analysis. However, for 148 hearing family members (139 island residents), we located birthdates and marriage dates; hence we were able to calculate ages at first marriage for those individuals.

From the set of hearing family members of deaf islanders, we identified 38 hearing island residents who had at least one deaf parent; another 18 deaf-parented hearing individuals never lived on the island. We also identified 51 younger siblings of deaf islanders; seven individuals were deaf-parented and younger than their deaf sibling(s). We created a third database in which we recorded information about deaf-parented individuals and younger siblings, including the dates during which they were resident on the island. In many cases, exact dates for periods of

island residence are difficult to determine, although reasonable inferences can typically be made based on the available genealogical and historical information. We make our inferences explicit in our databases.[19]

Distinguishing Between the Genetic Population of Carriers and the Signing Community

Here we describe the known origins of Vineyard deafness among the island population of European descent. We discuss the potential genetic carriers of Vineyard deafness in England and their links to New England. We argue that these populations should be considered separately from the signing community which arose on Martha's Vineyard.

The Origin of Vineyard Deafness

Based on the history of the English settlement of Martha's Vineyard, Groce (1985) surmised that the County of Kent, England may have been the origin of the autosomal recessive trait that caused deafness on the island. We are unaware of any research that has attempted to study the genetic etiology of Vineyard deafness. In general, a mutation (c.35delG-p.Gly12ValfsTer2) of the gap junction beta 2 (GJB2) gene (also called the connexin 26 gene; e.g., Nance, 2004) is most commonly associated with recessive non-syndromic sensorineural hearing loss among Europeans (Janecke et al., 2002; Aboagye et al., 2023). Different mutations of the same GJB2 gene have been implicated in populations in which other village signing communities have arisen. Scott et al. (1995) identified a mutation of GJB2 in the deaf population of Al-Sayyid, Israel. Brobby, Müller-Myhsok, and Horstmann (1998) identified a different mutation of GJB2 (c.427 C>T-p.Arg143Trp; see Aboagye et al., 2023) in the deaf population of Adamorobe, Ghana. That mutation may be widespread in Ghana: Hamelman et al. (2001, cited in Nyst, 2007) found it in 90% of a sample of 121 deaf Ghanaians from various parts of that country.

There appears to be no reason to suspect that Vineyard deafness originated in Kent. Aboagye et al. (2023) found that 10–20% of cases of congenital hearing loss among American Caucasians with origins in northern or southern Europe were attributable to the 35delG mutation.

Gasparini et al. (2000) found carrier frequencies of 1 in 35 in southern Europe and 1 in 79 in central and northern Europe. Thus, given the genetic bottleneck that can occur when a population moves to an island, it seems possible that other reproductively-isolated populations of European origin could, under similar circumstances, have experienced a comparable rate of deaf births to that seen on Martha's Vineyard.

Carriers of Vineyard Deafness

As noted, Groce (1985) argued that the recessive gene for Vineyard deafness was brought to Martha's Vineyard by English settlers from Kent (via intermediate settlements in Scituate, MA and on Cape Cod). She named three families in particular: the Lamberts, Skiffes, and Tiltons. Jonathan Lumbert and his children, Ebenezer and Buelah, are thought to have been the first deaf island residents (Banks, 1911b, p. 53). According to Groce (p. 25), nine deaf islanders were descendants of Jonathan. She drew on the earlier work of Bell, who traced the genealogies of many deaf islanders to early English settlers in New England, such as James Skiffe (also Skiff) and Samuel Tilton;[20] these two men are found in the lineages of numerous deaf islanders. In his notes on Skiffe, Bell reported the following (see Figure S1): "Of the known descendants of James Skiff 54 are deaf & dumb; and 8 deaf at an early age Total 62 deaf descendants." These 62 deaf individuals are all found in Bell's list of 72 "Deaf-Mutes of Martha's Vineyard"; see section Alexander Graham Bell's List of 72 Deaf Individuals. Later, Groce (p. 25) reported that Skiffe "appeared in sixty-three of the pedigrees" of the 72 Vineyard deaf.[21] It is possible that Skiffe's son, James Skiffe Jr. (who likely moved to the island in the late 1660s; Banks, 1911b), James Jr.'s wife Sarah Barnard,[22] Samuel Tilton, and Tilton's wife Hannah Moulton were carriers of the recessive gene for deafness.

<Insert Figure S1 here.>

One problem with Groce's hypothesized origin of Vineyard deafness is that many of these progenitors of island lineages into which deaf individuals would be born may not have come from Kent. According to Banks (1925, p. 229), the Lumbert family was likely from the county of Somerset. Other sources indicate that Jonathan Lumbert's grandfather migrated to Massachusetts from Thorncombe in the county of Dorset (or archaic Dorsetshire; Anderson, 1995, p. 1194), which lies on the border with Somerset. James Skiffe reportedly came to Massachusetts from London (Pierson, 1895)—though Banks (1911b, p. 71) noted a tenuous connection to Kent, namely, that there was at least one family of that surname living in Kent in 1609. Sarah Barnard's father Robert is thought to have come from Suffolk (see Hinchman, 1896); her mother may have come from Somerset, but this is uncertain.[23] Samuel Tilton was the son of William Tilton, who came with his wife to Massachusetts from Warwickshire, England (Jones, 1997). Hannah Moulton's ancestors apparently came to New Hampshire from Norfolk (Banks, 1911b, p. 39). Thus, these ancestors of many deaf islanders had no definite connection to Kent. See Kitzel (2013) for a detailed exploration of genealogical links between deaf individuals in Kent and New England.

Whatever its geographic origin, Vineyard deafness was a trait found only in the population descended from English settlers of New England. Bell's notes trace the deaf descendants of James Skiffe to many parts of New England, whether or not they ever lived on Martha's Vineyard. Nancy and Abigail Dillingham, both teachers at 19th-century schools for the deaf (Edwards, 2012), were seventh-generation descendants of Skiffe, according to Bell. However, they were born in western Massachusetts (in Lee), never lived on the Vineyard, and evidently never met other deaf individuals before enrolling at ASD (at ages 31 and 17, respectively). While attending ASD, Nancy Dillingham wrote about her life and her sister's prior

to coming to Hartford: "I supposed that my deaf and dumb sister and I were the only deaf and dumb in this world, and that all could hear and speak" (ASD, 1824, p. 32; cited in Edwards, 2012, p. 53).[24] Similarly, seven deaf children of Lemuel Newcomb (1782–1825; cf. Lane et al., 2011)[25] were born in Sandwich, MA and never lived on the Vineyard, but they too are included in Bell's notes because they were seventh-generation descendants of Skiffe. All told, 27 of the deaf individuals in Bell's notes on the descendants of James Skiffe were never Vineyard residents.[26] Thus, although these individuals may have formed part of the genetic population of carriers of Vineyard deafness, they were not members of the Vineyard signing community.

Linguistic Links Between the Vineyard, the Mainland, and the County of Kent in England

Groce (1985, p. 73) believed, based on genealogical connections between populations in Kent and on Martha's Vineyard, that MVSL may have had roots in British Sign Language (BSL) or in some other "English sign language" that was brought to New England from Kent. Kentish settlers began arriving in Massachusetts in the 1630s (Anderson 1991). If MVSL was indeed descended from a sign language used in Kent, that language must have arisen before the 1630s, and at least part of the signing community in Kent must have emigrated to New England. On this account, MVSL must have been over 350 years old when Groce was writing.

However, no evidence has come to light showing that any deaf individuals—that is, the individuals most likely to have formed the core of a signing community—emigrated from Kent to New England (Stone & Woll, 2008; Schembri et al., 2010; Kitzel, 2013). While it is possible that some hearing immigrants from Kent had been part of a signing community there, and that these hearing individuals continued to use a sign language from their arrival in Massachusetts in the 1630s until Jonathan Lumbert's birth some 20 years later, this account is purely speculative.

Other scholars (e.g., Bahan & Nash, 1995; Lane et al., 2000; Nash, 2015) have echoed Groce's hypothesis about a linguistic connection to Kent. The evidence has rested on one lexical comparison of MVSL with ASL and BSL in Bahan and Nash (1995); the methods and results reported there are explained in greater detail in Nash (2015). Of the comparison with BSL, Nash reported her results in two parts: (i) She compared her corpus of MVSL with a BSL dictionary, yielding 34 pairs of comparable signs, of which 13 were found to be "identical." Next, (ii) "a hearing native British signer" observed videos in Nash's corpus and "identified" 83 of 208 MVSL signs. Using these methods, MVSL and BSL were found to be 38–40% similar. In the comparison with ASL, Nash and her colleagues used a different methodology. Nash (p. 613) reported that "members of the New England Sign Language Society" determined that 46 of 208 MVSL signs "were recognized as ASL cognates." It is unclear whether the results of these two comparisons (i.e., MVSL-BSL vs. MVSL-ASL) are themselves comparable. The mere identification of MVSL signs, which could be formationally similar to BSL signs for many reasons including iconicity (see Guerra Currie, Meier, & Walters, 2002, for pertinent discussion), might have yielded a greater number of false positives (i.e., signs thought to be historically related but which are not) compared to the determination of "cognacy" by a group of linguistically-trained scholars.

In a recent analysis, Orfila (2023) found a higher rate of similarity (65%) in a comparison of MVSL to ASL than of MVSL to BSL (49%). According to Orfila, the similarity between MVSL and BSL can be explained by the similarity between ASL and BSL. That is, the signs in MVSL that are shared with BSL are also shared with ASL. Thus, in our view, evidence for a linguistic connection between BSL and MVSL is scant.

Alexander Graham Bell's List of 72 Deaf Individuals

Here we reproduce Alexander Graham Bell's list of 72 deaf individuals who were either born on the island (44; see lines 1–44 in Figure S2) or who lived off-island and had island ancestry (28; lines 50–77 in Figure S2); the list also includes six deaf individuals from off-island who married one of the 44 deaf island residents (lines 91–96 in Figure S2). Names in parentheses were represented in Bell's list with ditto marks (i.e., double apostrophes); these were apparently used for family members of the same generation. The following text is otherwise a verbatim transcription of Bell's handwritten list; an image of the list appears in Figure S2 below.

Deaf-Mutes of Martha's Vineyard

Deaf-Mutes of Vineyard Ancestry residing in the Vineyard

1. Butler, Andrew
2. Hammett, Caroline C.
3. (Hammett), Mary Olive
4. Harlock, Sarah
5. Lambert, Prudence D.
6. Luce, Almira G.
7. (Luce), Reliance
8. Luce, Catherine C.
9. Luce, Chas. H.
10. (Luce), Israel
11. Luce, Harry C.
12. Lumbert, Beulah
13. (Lumbert), Ebenezer

14. Lumbert, Jonathan

15. Mayhew, Alfred

16. (Mayhew), Elijah

17. (Mayhew), Benj.

18. (Mayhew), Ruby

19. (Mayhew), Love

20. Mayhew, Jerusha

21. (Mayhew), Wadsworth

22. Mayhew, Benj.

23. (Mayhew), Jared

24. Mayhew, Jonathan

25. (Mayhew), Mercy

26. Pease, George Corliss

27. Skiff, Alice M.

28. Skiff, Hannah

29. Skiff, Rebecca

30. Smith, Freeman N.

31. (Smith), Mary Brown

32. Smith, Lavina

33. Smith, Mary

34. (Smith), Sally

35. Tilton, Deidamia

36. (Tilton), Franklin

37. (Tilton), Zeno
38. Trask, Geo. West
39. West, Benj.
40. (West), Deidamia J.
41. (West), George Jr.
42. (West), Joseph E.T.
43. (West), Rebecca T.
44. West, Eva Sabrina

Deaf-Mutes of Vineyard ancestry (not residing in the Vineyard)

1. Allen, Josiah
2. (Allen), David L.
3. (Allen), Sally
4. (Allen), Mary
5. (Allen), Rebecca
6. Brown, Thos. L.
7. Dillingham, Abigail
8. (Dillingham), Nancy
9. Glidden, Annie
10. (Glidden), Mary
11. (Glidden), Caroline
12. (Glidden), George
13. Lovejoy, Charles
14. (Lovejoy), Hartwell

15. Lovejoy, Hartwell
16. (Lovejoy), Sarah
17. (Lovejoy), Emma
18. Newcomb, Jane Anderson
19. (Newcomb), John Whitman
20. (Newcomb), Josiah Sturgis
21. (Newcomb), William
22. (Newcomb), Abigail Nye
23. (Newcomb), Stephen Bassett
24. (Newcomb), Ellen Goodwin
25. Norton, Frances
26. (Norton), Hugh
27. Smith, Elijah
28. (Smith), Hannah

Deaf-Mutes who have married Martha's Vineyard deaf-mutes

1. Brown, Benj. K.
2. Brown, Thos.
3. Closson, Harriet
4. Foster, Sarah
5. Rogers, Sabrina
6. Trask, Eugene

Total number of deaf-mutes known to be descended from Martha's Vineyard people (Aug. 23, 1887) = 72 — all from Chilmark stock.

<Insert Figure S2 here.>

Jonathan Lumbert

Who was Jonathan Lumbert? Groce's (1985) account of the transmission of a sign language between Kent and Martha's Vineyard via Scituate, MA and Cape Cod greatly depends on her inferences about Jonathan Lumbert (or Lambert). Groce (p.71) argued that there must have been a widely-used sign language on the island that preceded Jonathan's arrival there in the early 1690s. She wrote that a sign language "must have existed prior to [the 18th century], because even for the first deaf islander, Jonathan Lambert, there seems to have been no language barrier." Many questions remain about the identity of this individual. Here we aim to describe the evidence as we now see it.

Was Jonathan Lumbert Deaf?

Judge Samuel Sewall met either the elder Jonathan or his son, who had the same name, on 5 April 1714 near what is now Lambert's Cove (i.e., Oukakemy Bay). The "Jonathan Lumbard" whom Sewall met was apparently fishing at night with a group that included a Wampanoag convert to Christianity. The key excerpt from the *Diary of Samuel Sewall* follows.

> "In our passage we were becalmed, and the Tide against us [so] that were 2½ hours getting over. Were fain to row to the west side of Oukakemy Bay, where we landed, the Sloop coming to anchor. Our Horses were forced to leap into the Sea. By that time had tackled them was duskish.
>
> Major Thaxter discovered some men and Horses, as he thought, upon the Beach, at a distance. When came to them found Thomas Paul, a Lame Indian, on Horseback with his Net on his shoulder, to catch Fish by Night. Upon my speaking to him to Pilot me, he left his Net and did it very well. We were ready to be offended that an Englishman, Jonathan Lumbard, in the Company spake not a

word to us, and it seems he is deaf and dumb. Got to Mr. Allen's a little before 9 at night." (Sewall, 1879, p. 432)

Figure S3 shows that "Jonathan Lumbard" was an insertion to this diary entry: The name appears in an interlinear position above a carat symbol which appears after the word "Englishman". We do not know how much time elapsed between the encounter described in the diary entry, the composition of the entry, and the insertion of Jonathan Lumbard's name.

<Insert Figure S3 here.>

We do not know whether Sewall's description—"it seems he is deaf and dumb"—referred to the elder Jonathan (then 56 years old) or the younger Jonathan (29). Who was likelier to have been fishing at night? We do not know. In either case, according to Sewall, one of them was deaf.

We know of no other references to the deafness of either Jonathan in any records, even though both appear frequently in the *Tisbury Town Records* (Swift & Cleveland, 1903). A few examples from those records follow. (Spelling, capitalization, and punctuation are reproduced as in the original inside quotations.)

- 6 February 1694/5: The elder "Jonathan Lumberd" was chosen (along with 5 other men) "to drive sheep after five dayes for to shere said sheep and mark and Cut Lambs" (p. 26).
- 26 March 1722: The elder "Jonathan Lumbert" (along with two other men) "ware Chosen gran Jere men for the Insuing year"; and the younger "Jonathan Lumbert Juner" was voted to "sarve Constable for the Insuing year" (p. 81).
- March 1723/4: The elder Jonathan was chosen as "modrater" for a "Leagal Town meeting," where it was decided that he (along with one other) would "take all propper means to supply the Town with A sutable minister to preach to sd Town" (p. 82).

- 21 February 1726/7: In the capacity mentioned in the preceding point, the elder Jonathan acted as the town's "faithfull messenger" to deliver the town's offer of employment to the "Reverend Mr. Nathanael Hancock Preacher of the Gospel" (p. 91).
- 17 March 1734/5: The younger Jonathan and two others were chosen to serve as selectmen (p. 100).

Evidently, both Jonathans were active in Tisbury's civic affairs. The elder Jonathan's integration into island society formed the basis for Groce's inference about the widespread use of sign language. This inference seems reasonable whether we assume that the elder or the younger Jonathan was deaf. After all, how could the younger Jonathan have served as selectman, a Tisbury municipal official, if others in the town were unable to sign? Relatedly, how could the elder Jonathan have moderated a town meeting? And how could he have served as the town's "faithfull messenger" to Reverend Hancock if he had been unable to communicate well with other islanders? Importantly, Hancock was not an islander by birth; he was born in Cambridge, MA, graduated from Harvard in 1721, and lived on the mainland in Woburn, MA as late as 1723 (Banks, 1911). Thus, the elder Jonathan was commissioned by the town to communicate important business to a Harvard-educated mainlander and perhaps to travel to Woburn.

As Groce emphasized, Lumbert's case is fascinating. More, if one of the Jonathans was indeed deaf, his case would be of great significance to Deaf history. To be elected as a Tisbury grand juror, Jonathan must have been literate in English. How did he become literate prior to the advent of schools for the deaf? We know of extremely few potentially parallel cases from that time period and prior to it.[27] Fernández Navarrete (1526–1579) was a painter whose intelligence, according to Plann (1993, p. 9), "was renowned at the Spanish [monarch Phillip II's] court"; he was at least partly literate, "able to write and sign his name," according to a self-report (cited in

Plann, 1997, p. 20). Navarrete, like the deaf offspring of other 16th-century Spanish aristocrats, was sent to a monastery at a young age; this practice stemmed in part from their families wishes to “conceal” their deaf children from public view (Plann, 1997, p. 13). However, Navarrete’s success, and that of the deaf students of Fray Pedro Ponce de Léon, was celebrated across Europe because it was assumed that prelingually deaf individuals “were inherently ineducable and that they could not learn to speak” (Plann, 1993, p. 6). In this light, Groce’s (1985, p. 81) conclusion about Lumbert appears to be an understatement: “To serve as the master of a vessel, to marry, to raise a large family and rise to a respected position in the community—all indicate that Lambert functioned effectively in Barnstable and Vineyard society.”

We wish, however, that we had more confidence that either Jonathan was indeed deaf. Despite intense interest among Enlightenment thinkers in the relationship of gesture to thought (Bacon, 1605/1893; Bulwer, 1644), despite multitudinous primary sources produced in colonial New England (e.g., Mather, 1684)[28] and voluminous research on the period (e.g., Anderson, 1991; Bremer, 1995), and despite recent research on pre-Revolutionary deaf Americans (e.g., Carty, Macready, and Sayers, 2009; Lang, 2007) and on deaf education in the 16th and 17th centuries (e.g., Plann, 1997; Van Cleve, 1993), there are no known references to the successful education of Jonathan Lumbert in 17th-century Massachusetts, to the service of a prelingually deaf individual as a municipal official, or to the extremely atypical sort of signing society understood by Groce to have existed on the island and perhaps on Cape Cod.

Perhaps Judge Sewall was mistaken and the fisherman whom he met was Jonathan Lumbert’s son, Ebenezer; or perhaps the elder Jonathan was late-deafened. Perhaps the offended Sewall’s comment was intended as an insult to Jonathan. Unfortunately, the historical record has thus far yielded only Sewall’s one sentence about Jonathan’s deafness.

Was Jonathan Lumbert a Ship's Captain?

In the previous quotation, Groce (1985) refers to Lumbert as the "master of a vessel." This reference can be traced back to Banks (1911, p. 53): "In 1695, Jonathan Lambert, master of the Brigantine *Tyral* was despatched to Quebec to bring back prisoners from that place. This may be our early settler." Note that Banks apparently misspelled the name of the ship as *Tyral*, instead of the correct *Tryal* (as in the modern spelling "trial"). See instructions (dated 2 August 1695) from the Lt. Governor of the Massachusetts Bay colony that mention Jonathan Lambert as "Master" of the "Briganteen Tryal" (Baxter, 1897, p. 421). Although Banks was apparently uncertain about whether this ship's captain and the deaf resident of Tisbury were one and the same person, Groce (p. 80) concluded that this was "almost certainly the same man."

Importantly, nowhere in the *Tisbury Town Records* are the elder or younger Jonathan Lumbert referred to as "Captain" or "Master" or "mariner," even though ship's captains were typically identified by one of those titles. For example, "Captain Thomas Butler" is mentioned at a town meeting on 14 May 1708 (Swift & Cleveland, 1903, p. 56). On 8 March 1736/7, in the same town meeting in which Jonathan Lumbert Junior was chosen as selectman, "Capt Samll Cobb" was chosen as surveyor (Swift & Cleveland, p. 104).

As it turns out, Jonathan Lumbert of Tisbury had a similarly named contemporary who lived in Boston and who was a master mariner ("ship-master"). This Jonathan Lambert was born in December 1669 and died about 1710 (Belknap, 1918, p. 17). He was also the member of a sea-faring family from Salem, MA; there were three or four generations of Jonathan Lamberts from this family who captained ships between 1695 and the early 19th century. On 17 June 1706, Jonathan Lambert arrived in Boston from Barbados, according to the *Boston News-Letter*. On the same day, the *Tryal* was reported to be outward bound, under a different master, to Montserrat.

Thus, we know that Jonathan Lambert of Boston was a ship's captain who was seen in Boston on the same day that the *Tryal* was in harbor.

In our view, it is much more likely that the Jonathan Lambert referred to in the Lt. Governor's instructions of 1695 was the Jonathan Lambert of Boston who is known to have been a ship's captain. If the "deaf and dumb" Jonathan Lumbert of Tisbury had been a ship's captain between at least 1695 and 1706, it would be difficult to explain why he was never identified as such in the Tisbury town records.

Key Divergences in Our Analyses From Prior Scholarship

Our analyses diverge somewhat from the analyses of prior scholars, such as those of Groce (1983; 1985), Lane et al. (2000), and Lane et al. (2011). Here we aim to highlight the most consequential of these divergences and to reconcile them to the extent possible.

A key advantage of studying the Vineyard signing community in the 2020s compared to the 1970s and 1980s is greater access to genealogical records in online repositories such as Ancestry and easier access to relevant historical records that have been scanned by Google Books since roughly 2017. One challenge for us has been that we have had only partial access to the data analyzed by prior scholars—except perhaps for Alexander Graham Bell's and Harlan Lane's papers. Hence we have been unable to directly compare our data to theirs. In addition, Groce (1985) has explained that the practice in the field of anthropology in the 1980s was to give pseudonyms to the individuals in her study. Although we have identified the preponderance of the individuals whom she identified as deaf, we have been unable to identify a small number of the deaf individuals she mentioned in her 1983 dissertation and 1985 book; see below.

Perhaps the most consequential divergence between our findings and Groce's lies in our estimate of the size of the Vineyard signing community. We have identified 53 total deaf island residents, whereas Groce (1985, p. 3) identified "at least 72." Notably, her figure is the same as the figure found in Bell's list of 72 "Deaf-Mutes of Martha's Vineyard"; see the section Alexander Graham Bell's List of 72 Deaf Individuals. However, recall that Bell's list is divided into three parts: "Deaf-Mutes of Vineyard Ancestry residing in the Vineyard," "Deaf-Mutes of Vineyard ancestry (not residing in the Vineyard)," and "Deaf-Mutes who have married Martha's Vineyard deaf-mutes." Thus, many of the 72 individuals in Bell's list never lived on the island; recall that some of those individuals, such as the Dillingham sisters, apparently did not know any

other deaf individuals before attending ASD. In our view, the only individuals who should be counted as part of the Vineyard signing community are those who lived on the island, interacted with other community members for an extended period, and were likely to have known the island's sign language.

Our figures differ from Groce's in other ways. Groce (1985, p. 22) reported that, of the 72 deaf individuals she identified, 34 were females and 29 were males, "in addition to 9 children listed as deaf for whom no records of sex are available." We counted 29 females and 24 males. We are not aware of any records that provided information about a child's deafness but not its sex. Perhaps that information came from Groce's oral history interviews. However, as we have shown, beginning with the 1817 selectmen reports, the historical record regarding deaf islanders is relatively complete and consistent. Thus, any deaf individuals who might have been identified in oral history interviews, but not in historical records, must have died before 1817. However, Bell would have been well-placed, from his vantage point at the end of the 19th century and with the assistance of Richard Pease, the island's genealogist, to discover information about deaf islanders via oral histories that would be lost to later researchers. Bell and Pease did not identify the nine deaf children referred to by Groce.

Compared to our analysis, Groce reported a higher peak of 19th-century deaf individuals. We report that the deaf population briefly peaked at 28 individuals in mid-1857 (see Fig. 2 in the main text). According to Groce (1985, p. 41), the deaf population peaked at 45 in the 1840s—a difference of 17 individuals. She also reported (p. 136) that there were 14 births of deaf children in the 1840s, whereas we count just 7.

As best we can tell, there is just one group of deaf individuals who are mentioned in Groce (1985) but whose identities we do not know. She (p. 83) reported the following about a

family of four deaf individuals purportedly living in Edgartown around the turn of the 19th century: "Benjamin E., who was deaf, and his hearing brother were lost bringing lumber over to the Island on a barge that sailed from New Bedford in the spring of 1805. He was survived by his deaf wife and two deaf sons." We have been unable to identify these individuals and the shipping accident.[29] It is possible that this family of deaf individuals did not live on the island. It seems certain that no deaf members of the family were resident on the island 12 years later, when the 1817 census of deaf individuals was taken. Edgartown did not return a census, presumably because there were no deaf individuals in the town then. There were also no deaf individuals reported in Edgartown in 1827 or 1830. In addition, the family does not appear in Bell's list of 72 individuals. Except for this family of four, Groce does not specifically refer to any deaf individuals whom we have been unable to identify.

Finally, it is possible that deaf individuals were undercounted on the island. Groce (1985) discussed the difficulties in working with census records pre-1850. In the section Sources and Methods, we described how we accounted for all deaf residents of Chilmark and Tisbury in the 1817 and 1827/8 selectmen reports, as well as all deaf islanders in the 1830 and 1840 federal censuses. Groce (p. 128) noted that, starting in 1850, "[a] national or state census was taken every five years, but it is quite conceivable that in that interval a young deaf child might die or the family might move off-island. Or a baby's deafness may not have been recognized at the first census." We agree, but such children may not have survived long enough, or resided on the island long enough, to have contributed significantly to the island's signing community.

There is, however, evidence that islanders sometimes recognized deafness at an early age and that deaf children, therefore, may not have been undercounted to the extent surmised by Groce. Prudence Lambert was 2 years old when the 1840 census was taken; see the section on

Methods above. She was the only deaf individual in her household and she had three older hearing siblings; hence her parents would not have been expecting a deaf child. Yet they recognized that she was deaf at the age of 2. Similarly, Mercy Mayhew, the first deaf child in her immediate family, was 2 years old at the time of the 1840 census; she too was recognized as deaf. Franklin Tilton was 3 in 1828 and was recognized as deaf. However, the deafness of some other children was not identified at young ages. Charles Luce was 11 months old at the time of the selectmen's report in January 1828; he was not identified as deaf in that report. However, he was noted as being deaf in the 1830 federal census, when he was 3 years old. Freeman Smith was 15 months old at the time of the 1850 census; he was not identified as deaf in that document. Thus, it is possible that deaf children who died or moved off-island before age 2 might not have been counted as deaf in any surviving historical record.

References

Adams, J. (1765). *The diary of John Adams*. Boston: Massachusetts Historical Society. https://www.masshist.org/digitaladams/archive/index

Anderson, R. C. (1995). *The Great Migration begins: Immigrants to New England, 1620–1633* (Vols. 1–3). Boston: New England Historic Genealogical Society.

Anderson, V. D. (1991). *New England's generation: The Great Migration and the formation of society and culture in the seventeenth century*. Cambridge: Cambridge University Press.

Anonymous. (1860, August 7). Letter to the Editor. *Vineyard Gazette.* https://vineyardgazette.com/news/1860/08/03/visit-vineyard

Anonymous. (1931, February 6). Interesting Vineyarders: Sophronia E. Hillman. *Vineyard Gazette*. https://vineyardgazette.com/news/1931/02/06/interesting-vineyarders-saphronia-e-hillman.

Bacon, F. (1893). *The advancement of learning*. London: Cassell & Company, Limited. https://www.gutenberg.org/files/5500/5500-h/5500-h.htm (Original work published 1605)

Banks, C. E. (1911). *The history of Martha's Vineyard Dukes County Massachusetts in three volumes: Vol. 2. Town annals*. Boston: George H. Dean.

Baxter, J. P. (1897). *Documentary history of the state of Maine* (Vol. 5). Portland, ME: The Maine Historical Society.

Belknap, H. W. (1918). *The Lambert family of Salem, Massachusetts*. Salem, MA: The Essex Institute. https://archive.org/stream/lambertfamilyofs00belk/lambertfamilyofs00belk_djvu.txt

Blake, K. (1996). "First in the path of the firemen": The fate of the 1890 population census. *Prologue Magazine*, *28*(1). https://www.archives.gov/publications/prologue/1996/spring/1890-census-1.

Bremer, F. J. (1995). *The Puritan experiment: New England society from Bradford to Edwards*. London, NH: University Press of New England.

Brobby, G. W., Müller-Myhsok, B., & Horstmann, R. D. (1998). Connexin 26 R143W mutation associated with recessive nonsyndromic sensorineural deafness in Africa. *The New England Journal of Medicine*, *338*, 548–550. https://www.nejm.org/doi/full/10.1056/NEJM199802193380813.

Bulwer, J. (1644). *Chirologia, or the natural language of the hand*. London: Tho. Harper. https://archive.org/details/gu_chirologianat00gent/mode/2up

Carty, B., Macready, S., & Sayers, E. E. (2009). "A grave and gracious woman": Deaf people and signed language in colonial New England. *Sign Language Studies*, *9*(3), 287–323.

Gasparini, P., Rabionet, R., Barbujani, G., Melchionda, S., Petersen, M., Brøndum-Nielsen, K., Metspalu, A., Oitmaa, E., Pisano, M., Fortina, P., Zelante, L., & Estivill, X. (2000). High carrier frequency of the 35delG deafness mutation in European populations. *European Journal of Human Genetics*, *8*, 19–23. https://doi.org/10.1038/sj.ejhg.5200406.

Gaw, A. C. (1907). *The legal status of the deaf*. Washington, DC: Gibson Brothers.

General Court of the Commonwealth of Massachusetts. (1819). *Resolve to ascertain the number of deaf and dumb persons, throughout the state*. Boston: Secretary of the Commonwealth. https://archive.org/details/actsresolvespass1519mass/page/418/mode/2up.

Gordon, Joseph C. (ed.). 1892. *Education of deaf children: Evidence of Edward Miner Gallaudet and Alexander Graham Bell*. Washington, DC: Volta Bureau.

Groce, N. E. (1983). *Hereditary deafness on the island of Martha's Vineyard: An ethnohistory of a genetic disorder* [Doctoral dissertation, Brown University]. Brown University ProQuest Dissertations Publishing. https://www.proquest.com/docview/303128979

Groce, N. E. (1985). *Everyone here spoke sign language: Hereditary deafness on Martha's Vineyard*. Cambridge, MA: Harvard University Press.

Guerra Currie, A.-M. P., Meier, R. P., & Walters, K. (2002). A cross-linguistic examination of the lexicons of four signed languages. In R. P. Meier, K. Cormier, & D. Quinto-Pozos (Eds.), *Modality and Structure in Signed and Spoken Languages* (pp. 224–236). Cambridge: Cambridge University Press.

Hamelman, C., Amedofu, G. K., Albrecht, K., Muntau, B., Gelhaus, A., Brobby, G. W., & Horstmann, R. D. (2001). Pattern of connexin 26 (GJB2) mutations causing sensorineural hearing impairment in Ghana. *Human Mutation*, *18*(1), 84–85. https://doi.org/10.1002/humu.1156

Harris, C. R., Millman, K. J., van der Walt, S. J., Gommers, R., Virtanen, P., Cournapeau, D., Wieser, E., Taylor, J., Berg, S., Smith, N. J., Kern, R., Picus, M., Hoyer, S., van Kerkwijk, M. H., Brett, M., Haldane, A., Fernández del Río, J., Wiebe, M., Peterson, P., … Oliphant, T. E. (2020). Array programming with NumPy. *Nature* 585. 357–362. https://doi.org/10.1038/s41586-020-2649-2

Hinchman, L. S. (1896). *Early settlers of Nantucket: Their associates and descendants*. Philadelphia: J.B. Lipincott Company.

Hunter, J. D. (2007). Matplotlib: A 2D graphics environment. *Computing in Science & Engineering*, *9*, 90–95. https://doi.ieeecomputersociety.org/10.1109/MCSE.2007.55

Janecke, A. R., Hirst-Stadlmann, A., Günther, B., Utermann, B., Müller, T., Löffler, J., Utermann, G., & Nekahm-Heis, D. (2002). Progressive hearing loss, and recurrent sudden sensorineural hearing loss associated with GJB2 mutations – phenotypic spectrum and

frequencies of GJB2 mutations in Austria. *Human Genetics*, *111*, 145–153. https://doi.org/10.1007/s00439-002-0762-y

Jones, W. H. (1997). *William Tilton: His English origins and some American descendants*. Bowie, MD: Heritage Press.

Jordahl, K., Van den Bossche, J., Fleischmann, M., Wasserman, J., McBride, J., Gerard, J., Tratner, J., Perry, M., Garcia Badaracco, A., Farmer, C., Hjelle, G. A., Snow, A. D., Cochran, M., Gillies, S., Culbertson, L., Bartos, M., Eubank, N., Bilogur, A., Rey, S. … Leblanc, F. (2020). GeoPandas: Python tools for geographic data (Version v0.8.1) [Computer software]. http://doi.org/10.5281/zenodo.3946761

Kitzel, M. E. (2013). Chasing ancestors: Searching for the roots of American Sign Language in the Kentish Weald, 1620–1851 [Doctoral dissertation, University of Sussex]. Sussex Research Online. http://sro.sussex.ac.uk/id/eprint/48877/.

Lane, H., Pillard, R. C., & French, M. (2000). Origins of the American deafworld: Assimilating and differentiating societies and their relation to genetic patterning. *Sign Language Studies*, *1*(1), 17–44. https://www.jstor.org/stable/26204927

Lane, H., Pillard, R. C., & Hedberg, U. (2011). *The people of the eye: Deaf ethnicity and ancestry*. Oxford: Oxford University Press.

Lang, H. G. (2007). Genesis of a community: The American deaf experience in the seventeenth and eighteenth centuries. In J. V. Van Cleve (Ed.), *The deaf history reader* (pp. 1–23). Washington, DC: Gallaudet University Press.

Mather, I. (1684). *An essay for the recording of illustrious providences: wherein an account is given of many remarkable and very memorable events, which have happened in this last age; especially in New-England*. Boston: Samuel Green for Joseph Browning.

McKinney, W. (2010). Data structures for statistical computing in Python. In S. van der Walt & J. Millman (Eds.), *Proceedings of the 9th Python in Science Conference* (pp. 56–61). Austin, TX: SciPy 2010.

New England Gallaudet Association of Deaf Mutes. (1854). *Proceedings of the constitutional committee*. Bradford, VT: Inquirer Office.

Nystrom, E. C., & Edwards, R. A. R. (in press). *Ordinary lives: Recovering deaf history through the American census*. Boston: University of Massachusetts Press.

Peet, H. P. (1857). *On the legal rights and responsibilities of the deaf and dumb*. Richmond, VA: C.H. Wynne's Steam-Power Presses.

Peterson, M. (2019). *The city-state of Boston: The rise and fall of an Atlantic power, 1630–1865*. Princeton: Princeton University Press.

Pierson, F. L. (1895). *The descendants of James Skiff of London, England and Sandwich, Mass., who died after 1688*. Amenia, NY: Walsh & Griffen, Printers.

Plann, S. (1993). Pedro Ponce de León: Myth and reality. In J. Vickrey Van Cleve (Ed.), *Deaf history unveiled: Interpretations from the new scholarship* (pp. 1–12). Washington, DC: Gallaudet University Press.

Plann, S. (1997). *A silent minority: Deaf education in Spain, 1550–1835*. Berkeley, CA: University of California Press.

Plotly Technologies Inc. (2015). *Collaborative data science* [Computer software]. Montréal: Plotly Technologies Inc.

Richardson, K. (2017). New evidence for Early Modern Ottoman Arabic and Turkish sign systems. *Sign Language Studies*, *17*(2), 172–192. https://doi.org/10.1353/sls.2017.0001

Schembri, A., Cormier, K., Johnston, T., McKee, D., McKee, R., & Woll, B. (2010). Sociolinguistic variation in British, Australian and New Zealand Sign Languages. In D. Brentari (Ed.), *Sign Languages* (pp. 476–498). Cambridge: Cambridge University Press.

Sewall, S. (1697/1888). The judge's confession. In E. C. Stedman & E. M. Hutchinson (Eds.), *A library of American literature: From the earliest settlement to the present time* (Vol. 2) (p. 188). New York: Charles L. Webster & Company. (Original work published 1697)

Sewall, S. (1700). *The selling of Joseph: A memorial*. Boston: Bartholomew Green and John Allen. https://www.masshist.org/database/53

Sewall, S. (1879). *Diary of Samuel Sewall, 1674–1729* (Vol. 2). Boston: Massachusetts Historical Society.

Stone, C., & Woll, B. (2008). Dumb O Jemmy and others: Deaf people, interpreters and the London courts in the eighteenth and nineteenth centuries. *Sign Language Studies, 8*(3), 226–240. https://www.jstor.org/stable/26190578

Swift, W. S., & Cleveland, J. W. (Eds.). (1903). *Records of the town of Tisbury, Mass., beginning June 29, 1669, and ending May 16, 1864*. Boston: Wright & Potter Printing Company.

Van Cleve, J. V. (Ed.). (1993). *Deaf history unveiled: Interpretations from the new scholarship*. Washington, DC: Gallaudet University Press.

Van Rossum, G., & Drake, F. L. (2009). *Python 3 reference manual*. Scotts Valley, CA: CreateSpace.

Virtanen, P., Gommers, R., Oliphant, T. E., Haberland, M., Reddy, T., Cournapeau, D., Burovski, E., Peterson, P., Weckesser, W., Bright, J., van der Walt, S. J., Brett, M., Wilson, J., Millman, K. J., Mayorov, N., Nelson, A. R. J., Jones, E., Kern, R., Larson, E. … SciPy 1.0

Contributors. (2020). SciPy 1.0: Fundamental algorithms for scientific computing in Python. *Nature Methods*, *17*(3), 261–272. https://doi.org/10.1038/s41592-019-0686-2

Walling, H. F. (1858). Map of the counties of Barnstable, Dukes and Nantucket, Massachusetts. Boston: D.R. Smith. https://www.geographicus.com/P/AntiqueMap/capecod-walling-1858

Wroth, L. K., & Zobel, H. B. (Eds.). (1965). *Legal papers of John Adams* (Vol. 1). Cambridge, MA: Harvard University Press.

Figure S1

Excerpt From Alexander Graham Bell's Notes on the Descendants of James Skiffe

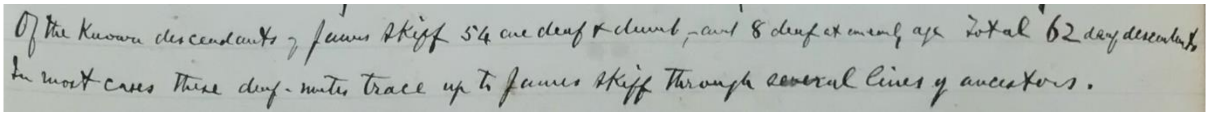
Of the known descendants of James Skiff 54 are deaf & dumb, and 8 deaf at early age. Total 62 deaf descendants.
In most cases these deaf-mutes trace up to James Skiff through several lines of ancestors.

Note. These notes are titled Deaf & Dumb descendants of James Skiff showing lines of descent, Box 7 of Bell's papers, Archives of the Alexander Graham Bell Association for the Deaf and Hard of Hearing.

Figure S2

Bell's (1887) List of 72 Deaf Individuals

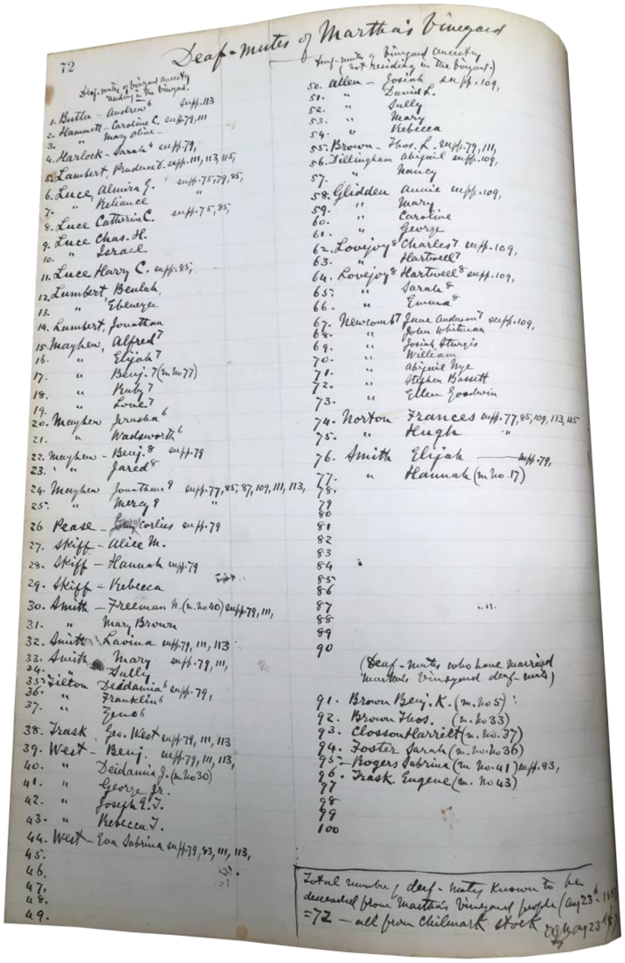

72

Deaf-mutes of Martha's Vineyard

Deaf-mutes of Vineyard ancestry residing in the Vineyard.

1. Butler – Andrew 6 suff. 113
2. Hammett – Caroline C. suff. 79, 111
3. " Mary Oline –
4. Harlock – Sarah 4 suff. 79,
5. Lambert, Prudence T. suff. 111, 113, 115,
6. Luce, Almira G. suff. 75, 79, 85,
7. " Reliance "
8. Luce Catharine C. suff. 75, 85,
9. Luce Chas. H.
10. " Israel
11. Luce Harry C. suff. 85,
12. Lumbert, Beulah,
13. " Ebenezer
14. Lumbert, Jonathan
15. Mayhew, Alfred 7
16. " Elijah 7
17. " Benj. 7 (m. No 77)
18. " Ruby 7
19. " Love 7
20. Mayhew Jerusha 6
21. " Wadsworth 6
22. Mayhew – Benj. 8 suff. 79
23. " Jared 8
24. Mayhew Jonathan 9 suff. 77, 85, 87, 109, 111, 113,
25. " Mercy 9 "
26. Pease – [illegible] Corliss suff. 79
27. Skiff – Alice M.
28. Skiff – Hannah suff. 79
29. Skiff – Rebecca
30. Smith – Freeman N. (m. No 40) suff. 79, 111,
31. " Mary Brown
32. Smith Lavina suff. 79, 111, 113
33. Smith Mary suff. 79, 111,
34. " Sally
35. Tilton Deidamia 6 suff. 79,
36. " Franklin 6
37. " Zeno 6
38. Trask Geo. West suff. 79, 111, 113
39. West – Benj. suff. 79, 111, 113,
40. " Deidamia J. (m. No 30)
41. " George Jr.
42. " Joseph Q. T.
43. " Rebecca T.
44. West – Eva Sabrina suff. 79, 83, 111, 113,
45.
46.
47.
48.
49.

Deaf-mutes of Vineyard ancestry (not residing in the Vineyard.)

50. Allen – Josiah suff. 109,
51. " David L.
52. " Sally
53. " Mary
54. " Rebecca
55. Brown – Thos. L. suff. 79, 111,
56. Dillingham Abigail suff. 109,
57. " Nancy
58. Glidden Annie suff. 109,
59. " Mary
60. " Caroline
61. " George
62. Lovejoy 8 Charles 7 suff. 109,
63. " Hartwell 7
64. Lovejoy 8 Hartwell 8 suff. 109,
65. " Sarah 8
66. " Emma 8
67. Newcomb 7 Jane Anderson 7 suff. 109,
68. " John Whitman
69. " Josiah Sturgis
70. " William
71. " Abigail Nye
72. " Stephen Bassett
73. " Ellen Goodwin
74. Norton Frances suff. 77, 85, 109, 113, 115
75. " Hugh "
76. Smith Elijah suff. 79,
77. " Hannah (m. No. 17)
78.
79
80
81
82
83
84
85
86
87
88
89
90

(Deaf-mutes who have married Martha's Vineyard deaf-mutes)

91. Brown Benj. K. (m. No 5)
92. Brown Thos. (m. No 33)
93. Closson Harriet (m. No. 37)
94. Foster Sarah (m. No. 36)
95. Rogers Sabrina (m. No. 41) suff. 83,
96. Trask. Eugene (m. No 43)
97
98
99
100

Total number of deaf-mutes known to be descended from Martha's Vineyard people (Aug 23d 1887) = 72 – all from Chilmark stock [illegible] Aug 23 1887

Note. From the Alexander Graham Bell Association's archives.

Figure S3

Excerpt From Sewall's (1879) Diary Referring to Jonathan Lumbard

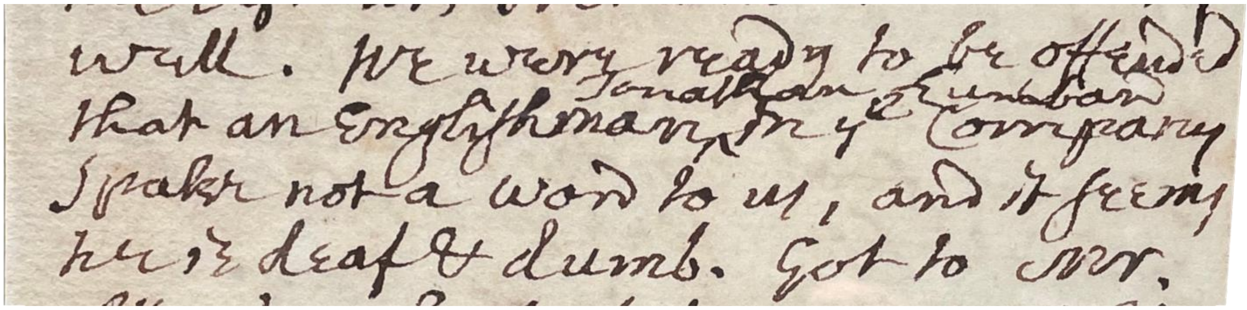
well. We were ready to be offended
that an Englishman Jonathan Lumbard in ye Company
Spake not a word to us, and it seems
he is deaf & dumb. Got to Mr.

Table S1

Individuals Identified as Deaf in Secondary Sources but Not in Primary Sources

Individual	Source
Andrew Butler	Bell
Sarah Harlock	Bell, Banks (p. 191), Groce (p. 42)
Jerusha Mayhew	Bell
Hannah Skiff	Bell
Rebecca Skiff	Bell
Benjamin Lambert	Lane (p. 238)
Jerusha Tilton	Lane (p. 98)
Zeno Tilton (1799–1817)	Lane (p. 240)
Four deaf individuals, possibly, in Edgartown in 1800	Groce (pp. 42, 83)

Note. Source column refers to Banks (1925), Bell's (1887) list of deaf individuals, Groce (1985), Lane et al. (2011).

Table S2

Deaf Residents of Chilmark According to the 1850 Federal Census

Deaf individual	Age in 1850
Benjamin Mayhew	65
Hannah Mayhew	41
Benjamin Mayhew	3
Elijah Mayhew	60
Ruby Mayhew	49
Love Mayhew	48
Alfred Mayhew	45
Deidamia West	31
Rebecca T. West	5
George West	1
Israel Luce	29
Charles Luce	23
Zeno Tilton	27
Franklin Tilton	25
Sarah W. Tilton	26
Prudence Lambert	12
Caroline C. Hammett	9

Note. Individuals between two horizontal lines were reported in the same household.

Table S3

Deaf Residents of Chilmark and Their Age Ranges According to the 1840 Federal Census

Named or inferred deaf individual	Actual birth	Actual age	Head of household (Relationship)	Reported age range
Benjamin Mayhew	8 Feb. 1785	55	Benjamin Mayhew	2 over age 25
Alfred Mayhew	30 Jan. 1805	35		
Elijah Mayhew	25 Jan. 1790	50	Elijah Mayhew	1 over age 25
Ruby Mayhew	19 June 1799	39	Lydia Mayhew	2 over age 25
Love Mayhew	27 Apr. 1802	38	(Mayhews' mother)	
Deidamia Tilton	16 July 1818	21	Samuel Tilton	3 ages 14–25
Zeno Tilton	13 June 1822	17	(Tiltons' father)	
Franklin Tilton	29 Aug. 1824	15		
Israel Luce	14 Feb. 1821	19	Ebenezer Luce	1 age 14–25
Charles Luce	14 Feb. 1827	13	(Luces' father)	1 under age 14
Prudence Lambert	24 Sep. 1837	2	Thomas Lambert (Lambert's father)	1 under age 14

Note. Individuals between two horizontal lines were reported in the same household.

Table S4

Deaf Residents of Chilmark and Their Age Ranges According to the 1830 Federal Census

Inferred deaf individual	Actual birth	Actual age	Head of household (Relationship)	Reported age range
Benjamin Mayhew	8 Feb. 1785	45	Lydia Mayhew	4 over age 25
Elijah Mayhew	25 Jan. 1790	40	(Mayhews' mother)	1 age 14–25
Ruby Mayhew	19 June 1799	29		
Love Mayhew	27 Apr. 1802	28		
Alfred Mayhew	30 Jan. 1805	25		
Deidamia Tilton	16 July 1818	11	Samuel Tilton	3 under age 14
Zeno Tilton	13 June 1822	7	(Tiltons' father)	
Franklin Tilton	29 Aug. 1824	5		
Israel Luce	14 Feb. 1821	9	Ebenezer Luce	2 under age 14
Charles Luce	14 Feb. 1827	3	(Luces' father)	
Sally Smith	1 Oct. 1806	24	Mayhew Smith	2 ages 14–25
Mary Smith	28 Feb. 1811	19	(Smiths' father)	

Note. Individuals between two horizontal lines were reported in the same household.

Table S5

Deaf Residents of Chilmark According to the Selectmen Report Dated 21 January 1828

Inferred deaf individual	Actual birth	Actual age	Reported age	Reported gender
Benjamin Mayhew	8 Feb. 1785	43	42	Male
Elijah Mayhew	25 Jan. 1790	37	37	Male
Ruby Mayhew	19 June 1799	28	28	Female
Love Mayhew	27 April 1802	25	25	Female
Alfred Mayhew	30 Jan. 1805	22	22	Male
Deidamia Tilton	16 July 1818	9	9	Female
Zeno Tilton	13 June 1822	5	5	Male
Franklin Tilton	29 Aug. 1824	3	3	Male
Israel Luce	14 Feb. 1821	6	6	Male
Sally Smith	1 Oct. 1806	21	21	Female
Mary Smith	28 Feb. 1811	16	16	Female

Table S6

Deaf Residents of Chilmark According to the Selectmen Report Dated 31 December 1817

Inferred deaf individual	Actual birth	Actual age	Reported age	Reported gender
Benjamin Mayhew	8 Feb. 1785	32	“the age of the youngest is about 14 years the oldest about 32 years”	3 m, 2 f
Elijah Mayhew	25 Jan. 1790	27		
Ruby Mayhew	19 June 1799	18		
Love Mayhew	27 Apr. 1802	15		
Alfred Mayhew	30 Jan. 1805	12		
Sally Smith	1 Oct. 1806	11	aged “about 7 years” and “about 12 years”	2 f
Mary Smith	28 Feb. 1811	6		

Note. Individuals between two horizontal lines were reported in the same household.

Notes

[1] See the Library of Congress' collections here: https://www.loc.gov/collections/alexander-graham-bell-papers/.

[2] Judge Samuel Sewall (1652–1730) is infamous for his role in the Salem witch trials (1692–1693). In 1697, before meeting Lumbard, Sewall (1697/1888, p. 188) evidently confessed his own guilt over his role in the trials: his confession reads that he "Desire[d] to take the Blame and shame of it, Asking pardon of men, And especially desiring prayers that God, who has an Unlimited Authority, would pardon that sin...." Later, Sewall (1700) wrote the first pamphlet against slavery that was published in Boston (Peterson, 2019).

[3] *Records of the Town of Tisbury, Mass* cover the period from 29 June 1669 to 16 May 1864.

[4] Massachusetts County, District and Probate Courts' Probate Records, Wills, Vol 1-3, 1690-1752 in ancestry.com's Massachusetts, U.S., Wills and Probate Records, 1635-1991.

[5] See Peet's (1857) *On the Legal Rights and Responsibilities of the Deaf and Dumb,* Gaw's (1907) *The Legal Status of the Deaf*, and Nystrom & Edwards (in press). English and American law in the 17th and 18th centuries gradually came to recognize the right of a deaf individual to make contracts and testaments using an interpreter and without proving literacy.

[6] Adams wrote the papers in Volume 1 between 1758 and 1778. Adams' papers are available through the Massachusetts Historical Society's website: https://www.masshist.org/publications/adams-papers/index.php/volume/ADMS-05-01.

[7] Massachusetts Archives, Series SC1/147x, Returns of the Deaf, 1817–1827.

[8] Turner's article in the *American Annals* did not precisely date his visit; he (1847, p. 28) wrote only: "Thinking there might be some special reason for the existence of so many cases [of

deafness] in that place, we took occasion while there some years since to inquire of the parents respecting it."

[9] Lydia Macombers "letter book" can be found at the Westport Historical Society's website: https://wpthistory.org/2014/11/lydia-macomber/. See also Nystrom & Edwards (in press).

[10] The 1890 federal census returns were almost entirely destroyed in a fire in 1921 (Blake, 1996). See https://www.archives.gov/publications/prologue/1996/spring/1890-census-1.html.

[11] The agreement, dated 11 December 1885, is available in the Alexander Graham Bell Family Papers at the Library of Congress.

[12] See also Bell's letters from the island to his wife, Mabel Hubbard Bell, which have been digitized by the Library of Congress: https://www.loc.gov/resource/magbell.03700217/?st=gallery.

[13] Bell's paper, *Upon the formation of a deaf variety of the human race*, is now rightfully regarded as wrongheaded and immoral. In it, he (1884, p. 46) concluded "the most promising method of lessening the evil [i.e., the formation of a 'deaf race'] appears to lie in the adoption of preventative measures.... [W]e should be guided by the following principle: (1.) *Determine the causes that promote intermarriages among the deaf and dumb; and* (2.) *remove them.*"

[14] Some evidence in Bell's papers indicates that he was uncertain about Hannah Skiff's ancestral lineage. He thought that Hannah's father was Charles Macy Skiff (b. ca. 1819). But Charles was the father of Alice Macy Skiff (1859–1937), who was deaf and is included in our analyses.

[15] Lane et al. may have mistaken this Zeno Tilton with another Zeno Tilton (1822–1881), who was indeed deaf.

[16] Inspection of Lane's papers suggests that he and his co-authors were uncertain about the attribution of deafness to Jerusha Tilton.

[17] In Moses Cromwell's household, the 1840 census reports one male in the 5-10 age range, one aged 15-20, two aged 20-30, and one 50-60. There were also two females 15-20 and one female 40-50.

[18] Almira (b.1832) and Reliance (b.1834) Luce were the daughters of Ulysses Luce and the granddaughters of Jabez Luce. Jabez and Ulysses lived in separate residences in 1840. Only one girl in Ulysses Luce's household was reported as deaf in 1840. It is therefore possible that the other daughter was living with her grandparents in 1840 and was reported there. However, for purposes of our analyses, we have included an additional unnamed deaf female who was born after the 1830 census and who died before the 1850 census.

[19] We will make these data available at: <placeholder for link>.

[20] Samuel Tilton (ca. 1638–1731) was an early settler on Martha's Vineyard; he moved there around 1673 (Banks, 1911b) after his 1662 marriage to Hannah Moulton (1645–1720).

[21] The source of the discrepancy between Bell's 62 individuals and Groce's 63 is unclear, in particular given that both scholars report to have identified 72 deaf individuals. It is possible that Groce identified Elizabeth Mary Newcomb, whom Bell had not identified (see footnote 49 below). If so, it is unclear why Groce did not report the total number of deaf individuals she identified as 73.

[22] Following his 1670 divorce from Elizabeth Tabor (Banks, 1911a, p. 474), James Skiffe, Jr. moved to Nantucket and remarried. His second wife, Sarah Barnard (1648?–1732),

may have been a carrier of Vineyard deafness; she was related, via her grandfather Thomas Barnard (1576–1621), to four ASD students who were born between 1814 and 1853; three of them were born on Nantucket.

[23] See Ancestry.com's online database of England, Select Births and Christenings, 1538–1975; FHL Film Number: 0097130, 0924137, 0924137 IT 2.

[24] Abigail Dillingham attended ASD from April 1817 to November 1819, and Nancy attended from October 1819 to April 1825. Thus, neither of them overlapped with the first three deaf students from Martha's Vineyard, who enrolled at the school in October 1825.

[25] Lemuel and Rebecca Newcomb had at least eight children (Lane et al., 2011). Three (William b.1812, Stephen b.1816, Elizabeth b.1820) died under age 1. Elizabeth Mary Newcomb was not identified in Bell's notes.

[26] Bell included Hannah Smith in his list of 28 off-island-born individuals because she was born in Maine. However, as Bell knew, she moved to the island after her 1843 marriage to Benjamin Mayhew (b.1785).

[27] See Richardson (2017) for details about deaf individuals in Ottoman palace courts as early as the 15th century.

[28] See the Massachusetts Historical Society's collection of pre-Revolutionary diaries: https://www.masshist.org/collection-guides/view/fa0240. See also the Increase Mather Papers: https://www.masshist.org/collection-guides/view/fa0239.

[29] In the immediately preceding sentence, Groce cites Joseph Thaxter's journal. We have searched that journal, and other sources, but have so far been unable to identify this family.
